# Edoxaban for stroke prevention in atrial fibrillation and factors associated with dosing: patient characteristics from the prospective observational ETNA-AF-China registry

**DOI:** 10.1038/s41598-024-51776-3

**Published:** 2024-02-02

**Authors:** Xueyuan Guo, Juan Du, Yang Yang, Mingxing Wu, Wenchao Ou, Xuebin Han, Zhifang Wang, Jing Jin, Ping Zhang, Zheng Zhang, Guoqin Chen, Mingzhi Long, Guotian Yin, Tong Liu, Xiaoyan Wang, Dongsheng Li, Manhua Chen, Yugang Dong, Chunlin Lai, Xuelian Zhang, Yuan Yi, Jing Xiang, Cathy Chen, Martin Unverdorben, Changsheng Ma, ETNA-AF-China Investigators, Yuehui Yin, Heng Qi, Zhisheng Jia, Xiaojun Ji, Yuqing Zhang, Xue Liang, Bing Deng, Jieyun Liu, Juan Ma, Cangsang Song, Huifang Feng, Suxin Luo, Jingfeng Wang, Lun Li, Yongqi Xiao, Junyou Cui, Xiang Cheng, Zheng Huang, Jiafu Wei, Jinfang Cheng, Haiyan Li, Jun Zhang, Weihong Jiang, Jie Liu, Xinwen Min, Peng Gao, Lianjun Gao, Zhouqing Huang, Ruxing Wang, Ying Li, Zebin Ye, Xubo Wang, Hengli Lai, Lihong Wang, Minli Zhang, Changqian Wang, Wei Mao, Xiaohua Chen, Zhirong Wang, Fang Liu, Xiaolin Xie, Zhenggui Xu, Bin Dong, Hesong Zeng, Jianqiang Peng, Lihua Zhang, Qingyan Zhao, Fucheng Liu, Caixia Guo, Lijiang Tang, Xianjin Li, Jing Zhou, Xinchun Yang, Xiaoshu Chen, Yajuan Liu, Tiebi Tong, Guanmin Tang, Jian Liu, Meise Lin, Bin Liu, Guosheng Fu, Weimin Wang, Qing Yang, Baopeng Tang, Boli Ran, Jianjiang Xu, Chunlin Yin, Pingzhen Yang, Fujie Yang, Ming Bai, Jian Zhang

**Affiliations:** 1 Department of Cardiology, Beijing Anzhen Hospital, Capital Medical University, Beijing, 100029 China; 2 Medical Department, Daiichi Sankyo (China) Holdings Co., Ltd, Shanghai, 200040 China; 3 Department of Cardiology, The Second Affiliated Hospital of Shenyang Medical College, Shenyang, 110004 Liaoning China; 4 Department of Cardiology, Xiangtan Central Hospital, Xiangtan, 411199 Hunan China; 5 Department of Cardiology, The Second Affiliated Hospital of Guangzhou Medical University, Guangzhou, 510260 Guangdong China; 6 Department of Cardiology, Shanxi Cardiovascular Hospital, Taiyuan, 030024 Shanxi China; 7 Deparment of Vasculocardiology, Xinxiang Central Hospital, Xinxiang, 453001 Henan China; 8 Department of Cardiology, The Fourth Hospital of Changsha, Changsha, 410006 Hunan China; 9 Department of Cardiology, Beijing Tsinghua Changgung Hospital, Beijing, 102218 Beijing China; 10 Department of Cardiology, QingPu Branch of Zhongshan Hospital Affiliated to Fudan University, Shanghai, 201799 Shanghai China; 11 Department of Cardiology, Guangzhou Panyu Central Hospital, Guangzhou, 511486 Guangdong China; 12 Department of Cardiology, The Second Affiliated Hospital of Nanjing Medical University, Nanjing, 210003 Jiangsu China; 13 Department of Cardiology, The Third Affiliated Hospital Of Xinxiang Medical University, Xinxiang, 453699 Henan China; 14 Department of Cardiology, The Second Hospital of Tianjin Medical University, Tianjin, 300211 Tianjin China; 15 Department of Cardiology, Affiliated Hospital of Jiangnan University, Wuxi, 214125 Jiangsu China; 16 Department of Cardiology, Wuhan Third Hospital, Wuhan, 430074 Hubei China; 17 Department of Cardiology, The Central Hospital of Wuhan, Wuhan, 430014 Hubei China; 18 Department of Cardiology, The First Affiliated Hospital, Sun Yat-Sen University, Guangzhou, 510080 Guangdong China; 19 Department of Cardiology, Shanxi Provincial People’s Hospital, Taiyuan, 043599 Shanxi China; 20 Department of Cardiology, Jilin Province People’s Hospital, Changchun, 130021 Jilin China; 21 Medical Department, Daiichi Sankyo Inc., Basking Ridge, NJ 07920-2311 USA; 22 The Second Affiliated Hospital of Chongqing Medical University, Chongqing, China; 23 The First Hospital of Changsha, Changsha, China; 24 The Fifth People’s Hospital of Jinan, Jinan, China; 25 Wenzhou Central Hospital, Wenzhou, China; 26 Nanjing Jiangning Hospital, Nanjing, China; 27 The Fifth Affiliated Hospital of Zhengzhou University, Zhengzhou, China; 28 Longhua Hospital Shanghai University of Traditional Chinese Medicine, Shanghai, China; 29 Kaifeng Central Hospital, Kaifeng, China; 30 The First Hospital of Kunming, Kunming, China; 31 Taiyuan Central Hospital of Shanxi Medical University, Taiyuan, China; 32 The First Affiliated Hospital of Chongqing Medical University, Chongqing, China; 33 Sun Yat-Sen Memorial Hospital, Sun Yat-Sen University, Guangzhou, China; 34 Wuhan Puai Hospital, Wuhan, China; 35 The Third People’s Hospital of Nanning, Nanning, China; 36 Jiangyin People’s Hospital, Wuxi, China; 37 Union Hospital Affiliated Withith Tongji Medical College of Huazhong University of Science and Technology, Wuhan, China; 38 The First Affiliated Hospital of Guangzhou Medical University, Guangzhou, China; 39 West China Hospital, Sichuan University, Chengdu, China; 40 Shanxi Bethune Hospital, Taiyuan, China; 41 Changzhou No.2 People’s Hospital, Changzhou, China; 42 Suzhou Municipal Hospital, Suzhou, China; 43 The Third Xiangya Hospital of Central South University, Changsha, China; 44 The First People’s Hospital of Nanning, Nanning, China; 45 Dongfeng General Hospital of Chinese Medicine, Shiyan, China; 46 Peking Union Medical College Hospital, Beijing, China; 47 The Fist Affiliated Hospital of Dalian Medical University, Dalian, China; 48 The First Affiliated Hospital of Wenzhou Medical University, Wenzhou, China; 49 Wuxi People’s Hospital, Wuxi, China; 50 Shanghai East Hospital, Tongji University, Shanghai, China; 51 Guangdong Second Provincial General Hospital, Guangzhou, China; 52 The Fourth Hospital of Jilin University, Changchun, China; 53 Jiangxi Provincial People’s Hospital, Nanchang, China; 54 Zhejiang Provincial People’s Hospital, Hangzhou, China; 55 Hebei Petro China Center Hospital, Langfang, China; 56 Shanghai Ninth People’s Hospital - Shanghai Jiaotong University School of Medicine, Shanghai, China; 57 Zhejiang Provincial Hospital of Chinese Medinice, Hangzhou, China; 58 The First People’s Hospital of Kunshan, Suzhou, China; 59 The Affiliated Hospital of Xuzhou Medical University, Xuzhou, China; 60 Aviation General Hospital, Beijing, China; 61 Hangzhou Third Hospital, Hangzhou, China; 62 Zhejiang Greentown Cardiovascular Hospital, Hangzhou, China; 63 Heping Hospital Affiliated to Changzhi Medical College, Changzhi, China; 64 Tongji Hospital Affiliated to Tongji Medical College of Huazhong University of Science and Technology, Wuhan, China; 65 Hunan Provincial People’s Hospital, Changsha, China; 66 The Second Affiliated Hospital of Zhengzhou University, Zhengzhou, China; 67 Renmin Hospital of Wuhan University - Hubei General Hospital, Wuhan, China; 68 The First Affiliated Hospital of Jinan University, Guangzhou, China; 69 Beijing Tongren Hospital, Beijing, China; 70 Zhejiang Hospital, Hangzhou, China; 71 Xuzhou Central Hospital, Xuzhou, China; 72 Peking University First Hospital, Beijing, China; 73 Beijing Chao-Yang Hospital Capital Medical University, Beijing, China; 74 Wenzhou People’s Hospital, Wenzhou, China; 75 Beijing Miyun District Hospital, Beijing, China; 76 China Shenyang Chest Hospital, Shenyang, China; 77 The First Hospital of Jiaxing, Jiaxing, China; 78 Guangzhou First People’s Hospital, Guangzhou, China; 79 Wenzhou Hospital of Traditional Chinese Medicine, Wenzhou, China; 80 The Second Hospital of Jilin Hospital, Changchun, China; 81 Sir Run Run Shaw Hospital - Zhejiang University School of Medicine, Hangzhou, China; 82 Heilongjiang Provincial Hospital, Haerbin, China; 83 The General Hospital of Tianjin Medical University, Tianjin, China; 84 The First Affiliated Hospital of Xinjiang Medical University, Urumchi, China; 85 Chongqing General Hospital, Chongqing, China; 86 The Second Hospital of Jiaxing, Jiaxing, China; 87 Xuanwu Hospital Capital Medical University, Beijing, China; 88 Zhujiang Hospital of Southern Medical University, Guangzhou, China; 89 Beijing Hospital, Beijing, China; 90 The First Hospital of Lanzhou University, Lanzhou, China; 91 Beijing Chest Hospital, Capital Medical University, Beijing, China

**Keywords:** Atrial fibrillation, Drug development

## Abstract

Real-world data on effectiveness and safety of a single non-vitamin K antagonist oral anticoagulant in the Chinese population with atrial fibrillation (AF) are limited. This study reports characteristics of patients treated with edoxaban and factors associated with dosing patterns from routine care in China. ETNA-AF-China (NCT04747496) is a multicentre, prospective, observational study enrolling edoxaban-treated patients from four economic regions with a targeted 2-year follow-up. Of the 4930 patients with AF (mean age: 70.2 ± 9.5 years; male, 57.1%), the mean creatinine clearance (CrCl), CHA_2_DS_2_-VASc, and HAS-BLED scores were 71.2 mL/min, 2.9, and 1.6. Overall, 6.4% of patients were perceived as frail by investigators. Available label dose reduction criteria (N = 4232) revealed that 3278 (77.5%) patients received recommended doses and 954 (22.5%) non-recommended doses. Northeast (53.0%) and West (43.1%) regions had the highest prescriptions of 60 mg and 30 mg recommended doses, respectively. Non-recommended 30 mg doses were more frequently prescribed in patients with antiplatelet use and history of heart failure than recommended 60 mg. Multivariate analysis identified advanced age as the strongest associated factor with non-recommended doses. Frailty had the strongest association with 30 mg except for age, and history of TIA was the most relevant factor associated with 60 mg. In conclusion, patients in the ETNA-AF-China study were predominantly aged 65 years and older, had mild-to-moderate renal impairment and good label adherence. Advanced age was associated with non-recommended doses, with frailty most common for non-recommended 30 mg and a history of TIA for the non-recommended 60 mg dose.

## Introduction

Atrial fibrillation (AF) is the most frequent chronic cardiac arrhythmia and is associated with increased morbidity, such as ischaemic stroke (9.48% vs. 2.26% in non-AF) and mortality^1^. Recent estimates in China have reported that 1.6% to 1.8% of adults are affected with AF, with marked regional variations in the prevalence, which was higher in the Eastern region (48.3%) and lesser in the Western region (16.9%)^2–4^. Studies observed that Chinese patients with AF had a variation in anticoagulant use, and underuse is reported to be predominant across medical institutions despite the severest disease burden of stroke^5–7^.

Globally, guidelines recommend non-vitamin K antagonist oral anticoagulants (NOACs) as the preferred option of reducing stroke risk in patients with AF^8^. Edoxaban is a direct oral anticoagulant indicated for stroke prevention in adult patients with nonvalvular atrial fibrillation (NVAF) who have one or more risk factors, including congestive heart failure (CHF), hypertension, age of ≥ 75 years, diabetes mellitus (DM) and prior stroke or transient ischaemic attack (TIA). The recommended dose of edoxaban is 60 mg once daily (OD) in AF patients, with dose reduction to 30 mg OD in patients who meet the label criteria^9^. Edoxaban was approved by the National Medical Products Administration (NMPA) based on the ENGAGE AF-TIMI 48 trial, which demonstrated non-inferiority of edoxaban to warfarin in preventing ischaemic stroke and systemic embolic events (SEEs), with a lower risk of bleeding events and cardiovascular (CV) deaths^10^. Real-world evidence from the Edoxaban Treatment in Routine Clinical Practice for Patients With Non Valvular Atrial Fibrillation (ETNA-AF) Global programme^11^, including ETNA-AF-Europe^12,13^, ETNA-AF-Japan^14,15^, and other Asian countries/regions^16^, has demonstrated effectiveness and safety of edoxaban in the treatment of a wide range of patient populations with AF. However, the benefits and risks of edoxaban in the real-world setting from Mainland of China are still unknown.

Furthermore, the recent European Society of Cardiology (ESC)/European Heart Rhythm Association (EHRA)/Asia Pacific Heart Rhythm Society (APHRS) guidelines and Journal of the American College of Cardiology statements emphasise the integration of the recommendation for patient-centred symptom management, CV and comorbidity management to improve prognosis^17,18^. In clinical practice, the prescription of NOACs is distinctly based on clinical characteristics^19^. The usage rate of non-recommended doses as per label recommendation was about 20% worldwide for AF treatment, and those patients faced poor outcomes, including higher mortality risk^20,21^. The medication adherence according to the label recommendation can be improved with a well-defined prescription pattern as well as early identification and management of independent associated factors.

The real-world, prospective, observational ETNA-AF-China registry is being conducted in AF patients treated with edoxaban from Mainland of China. While patients receive edoxaban in real-world clinical practice, the safety will be evaluated during a 2-year follow-up period. The primary objective of this study includes the evaluation of intracranial haemorrhage [ICH], adverse events [AEs] such as liver AE, CV, and all-cause mortality). This baseline analysis describes the characteristics of enrolled patients and explores the factors associated with edoxaban dosing prescription in clinical practice.

## Methods

**Figure 1:**
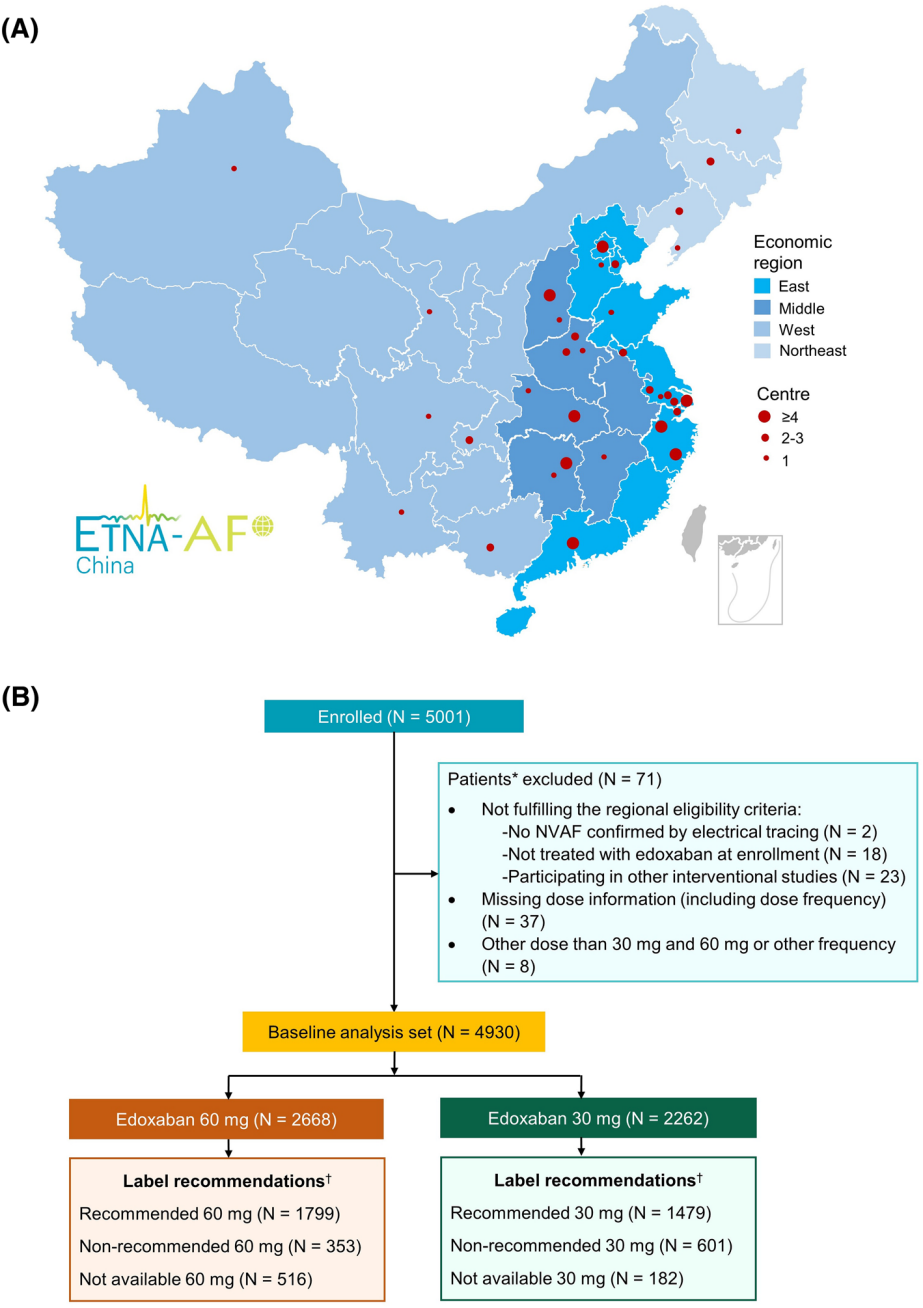
Overview of ETNA–AF–China study enrolment by (**A**) geographical distribution of centres, (**B**) flowchart. *A patient may fulfil one or more exclusion criteria; ^†^Patients with one or more of the following clinical factors were dose reduced: moderate or severe renal impairment (creatinine clearance (CrCl) ≤ 50 mL/min), body weight ≤ 60 kg or concomitant use of certain P-glycoprotein inhibitors listed in label and recorded at baseline. Not available category was considered on missing information for at least one potential dose reduction factor (i.e., renal impairment, body weight, P-glycoprotein inhibitors use), and no other reason fulfilled. CrCl, creatinine clearance; NVAF, nonvalvular atrial fibrillation.

ETNA-AF-China (ClinicalTrials.gov: NCT04747496) is a multicentre, non-interventional, prospective, observational study conducted in 89 centres from four economic regions (East, Middle, West and Northeast as sequence of GDP from high to low) across Mainland of China (Fig. 1A; Table S1). This study use the method of economic region division in Mainland of China developed by National Bureau of Statistics of China under the policy of coordinated development, in which Bejing, Tianjing, Hebei, Shanghai, Jiangsu, Zhejiang, Fujian, Shandong, Guangdong, Hainan consisting East; Shanxi, Anhui, Jiangxi, Henan, Hubei, Hunan consisting Middle; Inner Mongolia, Guangxi, Chongqing, Sichuan, Guizhou, Yunnan, Tibet, Shaanxi, Gansu, Qinghai, Ningxia, Xinjiang consisting West; Liaoning, Jilin, Heilongjiang consisting Northeast.

As part of the global initiative, comprising separate, non-interventional, prospective ETNA-AF registries in Europe, East Asia and Japan, ETNA-AF-China study is of similar design^22,23^. The study was approved by an institutional review board and Ethics Committee from Beijing Anzhen Hospital (No. 2019-058D), and all other clinical sites in compliance with the Declaration of Helsinki and Guidelines for Good Clinical Practice by International Conference for Harmonisation (ICH) and NMPA. All participants provided their written informed consent before participating in the study.

Eligible patients were unselected AF patients, treated with edoxaban at enrolment, and providing consent, and not simultaneously participating in any other interventional study. In the analysis, only patients treated with 60 mg or 30 mg OD edoxaban were included (Fig. 1B).

### Baseline data collection

Baseline data were collected through an electronic subject case report form (eCRF), which recorded demographic information, vital signs, renal and hepatic parameters, medical history, types and symptoms of AF, previous/concomitant medications, and administration status of edoxaban. The CHA_2_DS_2_-VASc and HAS-BLED scores were calculated based on the clinical characteristics. The values of creatinine clearance (CrCl) were calculated by the Cockcroft-Gault formula, and estimated glomerular filtration rate (eGFR) was normalized to 1.73 m^2^ of body surface area (BSA) using Du Bois and Du Bois^24^. Renal impairment stage was classified based on eGFR range according to the KDIGO guideline^25^. Frailty and fall risk of each person were generally recorded by investigators’ clinical judgement as the perceived aspect. The objective frailty was also determined using an adaptive modified frailty index (MFI)^26^, including the following variables: frequency of fatigue, dyspnea, or dizziness, history of diabetes mellitus (DM), Chronic obstructive pulmonary disease (COPD), Congestive heart failure (CHF), Myocardial infarction (MI), percutaneous coronary intervention (PCI), peripheral artery disease (PAD), transient ischaemic attack (TIA), or ischaemic stroke, and patients receiving treatment for hypertension. The index was calculated as ‘total number of variables present (each score as 1)/11 total variables’, where frailty was identified in a patient if the score was > 0.36^26,27^.

Associated factors between clinical characteristics and the edoxaban non-recommended dose were assessed. The recommended dose for edoxaban treatment is 60 mg OD as labelled and was reduced to 30 mg OD for patients who met at least 1 of the following criteria: (i) moderate-severe renal impairment (CrCl ≤ 50 mL/min), (ii) body weight ≤ 60 kg and (iii) concomitant use of label-listed P-glycoprotein (P-gp) inhibitors (Figure S1)^19^. Patients receiving non-recommended doses were referred to as not in line with label recommendation, i.e., 60 mg non-recommended as fulfilling at least one dose reduction criterion or 30 mg non-recommended without meeting any dose reduction criterion.

### Statistical analysis

Continuous variables are presented as mean ± standard deviation (SD), whereas categorical variables are presented as frequencies and percentages (N [%]). To compare the distribution of patient parameters between groups, the Mann–Whitney U test for ordinal and continuous variables or the chi-squared test for categorical variables was used, wherever applicable. To identify factors associated with edoxaban non-recommended treatment under the known dose reduction criteria, a multivariable logistic regression analysis was performed by stepwise method entering variables with *P* < 0.05 at univariate logistic regression analysis (under reduced criteria). Odds ratios (ORs) with 95% confidence intervals (CIs) and *P* values for the effect of each variable on edoxaban treatment patient groups were estimated. Missing data were not computed.

All statistical analyses were performed using R Studio Statistical Software Version 4.2.2 (R Foundation for Statistical Computing, Vienna, Austria). A 2‐sided *P* < 0.05 was considered statistically significant.

## Results

### Characteristics of overall population enrolled

A total of 5001 patients were enrolled in ETNA-AF-China study between February 2021 and October 2022, of which 71 patients were excluded due to not meeting eligibility criteria, missing information on edoxaban treatment and receiving doses other than 60 mg or 30 mg, or at a frequency other than OD. Of 4930 patients included, the percentage of patients from economic regions of China were as follows: East (*n* = 2459 [49.9%]), Northeast (*n* = 426 [8.6%]), Middle (*n* = 1645 [33.4%]) and West (*n* = 400 [8.1%]) (Fig. 1A and B).

The baseline characteristics of overall patient population (edoxaban 60 mg: *n* = 2668 [54.1%]; edoxaban 30 mg: *n* = 2262 [45.9%]) are summarised in Table 1. The mean patient age was 70.2 ± 9.5 years, with the largest (41.7%) proportion aged 65–74 years. The mean weight was 68.0 ± 12.6 kg, and mean body mass index (BMI) was 25.0 ± 3.7 kg/m^2^. The average calculated CrCl and eGFR reflecting renal function were 71.2 ± 27.7 mL/min and 58.9 ± 20.1 mL/min/1.73 m^2^, respectively. The calculated CHA_2_DS_2_-VASc and HAS-BLED mean scores were 2.9 ± 1.4 and 1.6 ± 0.9, respectively.

**Table 1:** Baseline demographics and clinical characteristics for overall population included in ETNA-AF-China.

	Total [N = 4930] (100%)	60 mg [N = 2668] (54.1%)	30 mg [N = 2262] (45.9%)
Male, n (%)	2815 (57.1%)	1824 (68.4%)	991 (43.8%)
Age (years), mean (SD)	70.2 (9.5)	67.6 (9.1)	73.4 (9.1)
By age sub-groups, n (%)			
< 65 years	1158 (23.5%)	829 (31.1%)	329 (14.5%)
65–74 years	2055 (41.7%)	1236 (46.3%)	819 (36.2%)
75–84 years	1481 (30.0%)	559 (21.0%)	922 (40.8%)
≥ 85 years	236 (4.8%)	44 (1.6%)	192 (8.5%)
Body weight (kg), mean (SD)	68.0 (12.6)	73.3 (11.2)	61.7 (11.3)
BMI (kg/m^2^), mean (SD)	25.0 (3.7)	26.2 (3.4)	23.5 (3.5)
SBP (mmHg), mean (SD)	126.5 (15.6)	127.1 (15.2)	125.7 (16.1)
DBP (mmHg), mean (SD)	76.7 (10.5)	77.8 (10.4)	75.4 (10.5)
(current) Smokers, n (%)	499 (10.1%)	356 (13.3%)	143 (6.3%)
No alcohol abuse, n (%)	4340 (88.0%)	2296 (86.1%)	2044 (90.4%)
(calc) CrCl^a^ (mL/min), mean (SD)	71.2 (27.7)	81.7 (26.8)	59.5 (23.7)
(calc) eGFR^b^ (mL/min/1.73 m^2^), mean (SD)	58.9 (20.1)	64.9 (18.9)	52.1 (19.2)
(calc) CHA_2_DS_2_-VASc Score^c^, mean (SD)	2.9 (1.4)	2.6 (1.3)	3.3 (1.4)
(calc) HAS-BLED Score^d^, mean (SD)	1.6 (0.9)	1.4 (1.0)	1.9 (0.8)
Perceived frailty, n (%)	316 (6.4%)	92 (3.45%)	224 (9.9%)
Risk of fall, n (%)	276 (5.6%)	76 (2.9%)	194 (8.6%)
Objective frailty^f^, n (%)	159 (3.2%)	72 (2.7%)	87 (3.9%)
LVEF categorised by 40%^g^			
< 40%	166 (3.4%)	81 (3.0%)	85 (3.8%)
≥ 40%	2400 (48.7%)	1285 (48.2%)	1115 (49.3%)
History of CV disease, n (%)			
Hypertension	3600 (73.0%)	2008 (75.3%)	1592 (70.4%)
Heart failure^h^	711 (14.4%)	325 (12.2%)	386 (17.1%)
Myocardial infarction	56 (1.1%)	28 (1.1%)	28 (1.2%)
Angina pectoris	176 (3.6%)	87 (3.3%)	89 (3.9%)
Valvular disease	251 (5.1%)	109 (4.1%)	142 (6.3%)
PAD	125 (2.5%)	66 (2.5%)	59 (2.6%)
History of diabetes mellitus, n (%)	1292 (26.2%)	744 (27.9%)	548 (24.2%)
History of COPD, n (%)	237 (4.8%)	111 (4.2%)	126 (5.6%)
History of Dyslipidaemia, n (%)	1226 (24.9%)	727 (27.3%)	499 (22.1%)
History of stroke and ICH, n (%)			
Ischaemic stroke	353 (7.2%)	173 (6.5%)	180 (8.0%)
Haemorrhagic stroke	33 (0.67%)	18 (0.67%)	15 (0.66%)
Stroke, unknown	64 (1.3%)	29 (1.1%)	35 (1.6%)
TIA	108 (2.2%)	58 (2.2%)	50 (2.2%)
ICH	35 (0.71%)	19 (0.71%)	16 (0.71%)
History of bleeding, n (%)			
Major	54 (1.1%)	30 (1.1%)	24 (1.1%)
CRNM	8 (0.16%)	2 (0.07%)	6 (0.27%)
GI bleeding	21 (0.43%)	6 (0.22%)	15 (0.66%)
Minor	61 (1.2%)	27 (1.0%)	34 (1.5%)
Chronic hepatic disease, n (%)	207 (4.2%)	107 (4.0%)	100 (4.4%)
Type of AF, n (%)			
Paroxysmal	1803 (36.6%)	931 (34.9%)	872 (38.6%)
Persistent	1604 (32.5%)	871 (32.7%)	733 (32.4%)
Long-standing persistent	836 (17.0%)	484 (18.1%)	352 (15.6%)
Permanent	670 (13.6%)	373 (14.0%)	297 (13.1%)
Time since first AF diagnosis (months), mean (SD)	54.8 (70.8)	56.2 (71.8)	53.1 (69.5)
Previous and concomitant medications, n (%)			
VKA	960 (19.5%)	544 (20.4%)	416 (18.4%)
NOAC (other)	2203 (44.7%)	1202 (45.1%)	1001 (44.3%)
Antiarrhythmics	2249 (45.6%)	1213 (45.5%)	1036 (45.8%)
Antiplatelet	723 (14.7%)	352 (13.2%)	371 (16.4%)

^a^creatinine clearance (CrCl) was estimated using the Cockcroft-Gault equation, N = 4004. ^b^eGFR was normalized to 1.73 m^2^ of BSA, N = 3937. ^c^CHA_2_DS_2_-VASc Score: CHF history, hypertension, elderly, diabetes mellitus, stroke (TIA, SEE) history, vascular disease (PAD, MI-STEMI, MI-NSTEMI), female; N = 4792. ^d^HAS-BLED Score: hypertension, abnormal renal function, abnormal liver function, stroke history, bleeding, elderly, drugs, alcohol abuse; N = 3991. ^e^Perceived frailty was left to discretion of the physician to categorise a patient as frail. ^f^Objective frailty assessed by a modified MFI score. ^g^LVEF data was recorded in 2566 patients. ^h^Heart failure (derived): if he/she has at least one of the following: CHF, ischaemic cardiomyopathy, LEVF < 40%, frequent dyspnea (≥ 1/day) without COPD and with at least one of the following: severe valvular heart disease, coronary heart disease, MI, valve replacement, documented hypertension treated with at least 3 drugs.

AF, atrial fibrillation; BMI, body mass index; Calc, calculated; CHF, congestive heart failure; COPD, chronic obstructive pulmonary disease; CrCl, creatinine clearance; CRNM, clinically relevant non-major; CV, Cardiovascular; DBP, diastolic blood pressure; eGFR, estimated glomerular filtration rate; GI, gastrointestinal; ICH, intracranial haemorrhage; LVEF, left ventricular ejection fraction; NOAC, non-vitamin K antagonist oral anticoagulant; PAD, peripheral artery disease; SBP, systolic blood pressure; SEE, systemic embolic event; TIA, transient ischaemic attack; VKA, vitamin K antagonist; SD, standard deviation.

Overall, 6.4% (*n* = 316) of patients were perceived frail as assessed by the investigators, 5.6% were considered at risk of fall. The percentage of objective frailty according to the MFI score was 3.2% (*n* = 159). The most common comorbidity was hypertension (73.0%), followed by DM (26.2%), dyslipidaemia (24.9%), and heart failure (HF [14.4%]). History of ischaemic stroke, TIA, and ICH occurred in 7.2%, 2.2%, and 0.71% of patients, whereas 1.1% reported a history of major bleeding. At the time of enrolment, the first diagnosis of AF was made on average (± SD) over 4.5 years (54.8 ± 70.8 months) ago with 36.6% presenting with paroxysmal and 32.5% with persistent AF. A higher proportion of patients with AF concomitant or switched from the previously taken anticoagulants to edoxaban (Vitamin K antagonists [VKA]: 19.5%; other NOACs: 44.7%; Table 1).

### Prescription patterns of edoxaban in patients with AF

**Figure 2:**
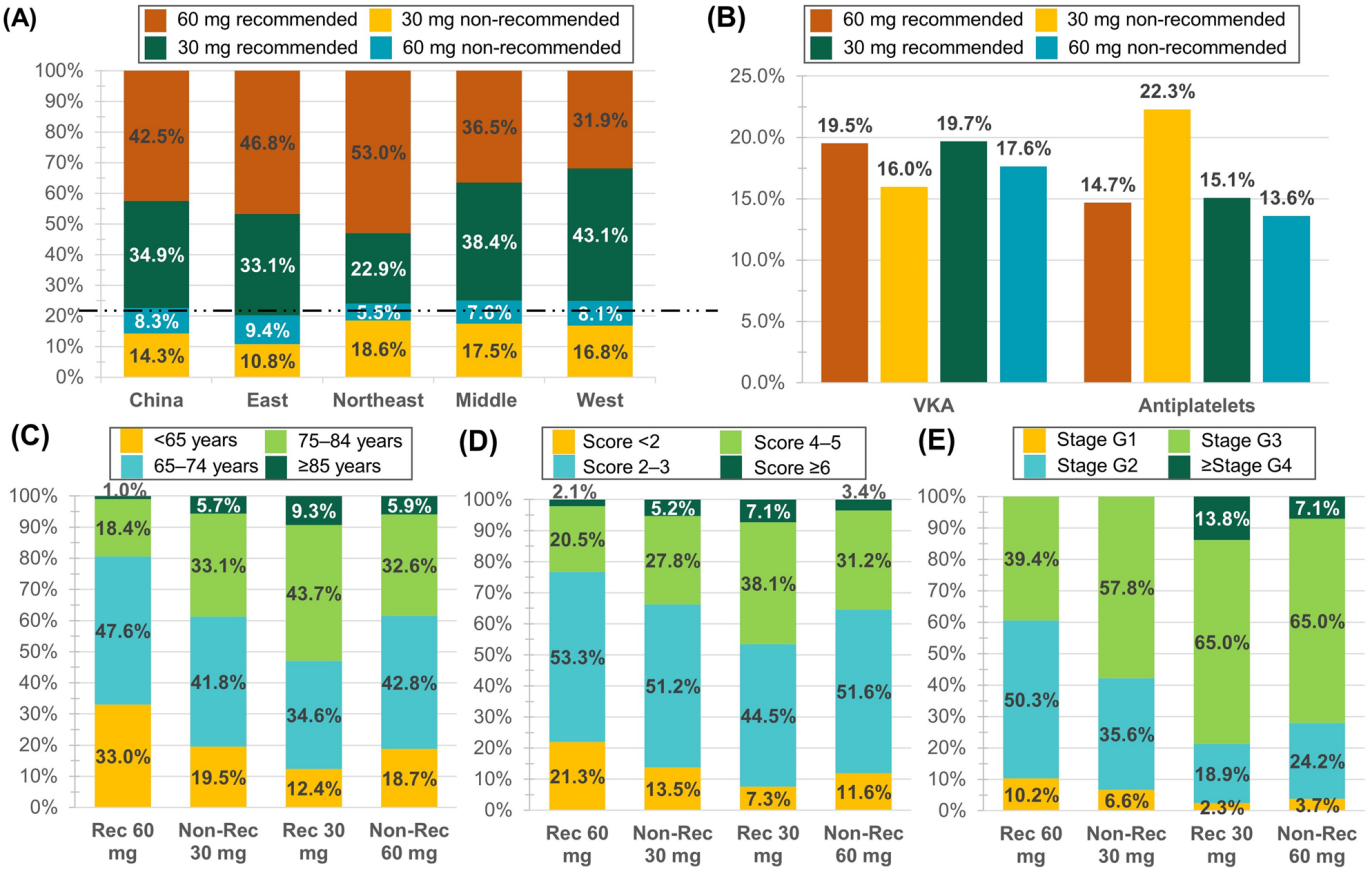
Prevalence of doses according to (**A**) economic regions, and percentage of (**B**) previous using antiarrhythmics or antiplatelets, distribution of (**C**) age, (**D**) CHA_2_DS_2_–VASc score and (**E**) renal impairment as label recommendation. Renal impairment stages: Stage G1 eGFR ≥ 90 mL/min/1.73 m^2^, Stage G2 eGFR 60–90 mL/min/1.73 m^2^, Stage G3 eGFR 30–60 mL/min/1.73 m^2^, Stage G4 eGFR 15–30 mL/min/1.73 m^2^, Stage G5 eGFR < 15 mL/min/1.73 m^2^. eGFR, estimated glomerular filtration rate.

Patients with dose reduction criteria available (*n* = 4232/4930) were further stratified to recommended (*n* = 3278 [77.5%]; 60 mg recommended: *n* = 1799 [42.5%]; 30 mg recommended: *n* = 1479 [34.9%]) and non-recommended (*n* = 954 [22.5%]; 60 mg non-recommended: *n* = 353 [8.3%]; 30 mg non-recommended: *n* = 601 [14.2%]) dosing according to the label criteria (Fig. 1; Figure S1). The general adherence rate to label recommendation was high with similar percentages across economic regions (Fig. 2A). The Northeast region has the highest percentage of recommended 60 mg doses (53.0% vs 46.8% in East, 36.5% in Middle, 31.9% in West), and the lowest percentage of non-recommended 60 mg doses (5.5% vs 7.6% in Middle, 8.1% in West, 9.4% in East). As for 30 mg, the West region counted as the highest percentage of the recommended dose (43.1% vs 38.4% in Middle, 33.1% in East, 22.9% in Northeast); the highest percentage of the non-recommended dose was observed in Northeast followed by Middle, West, and the East region (18.6%, 17.5%, 16.8%, 10.8%).

**Figure 3:**
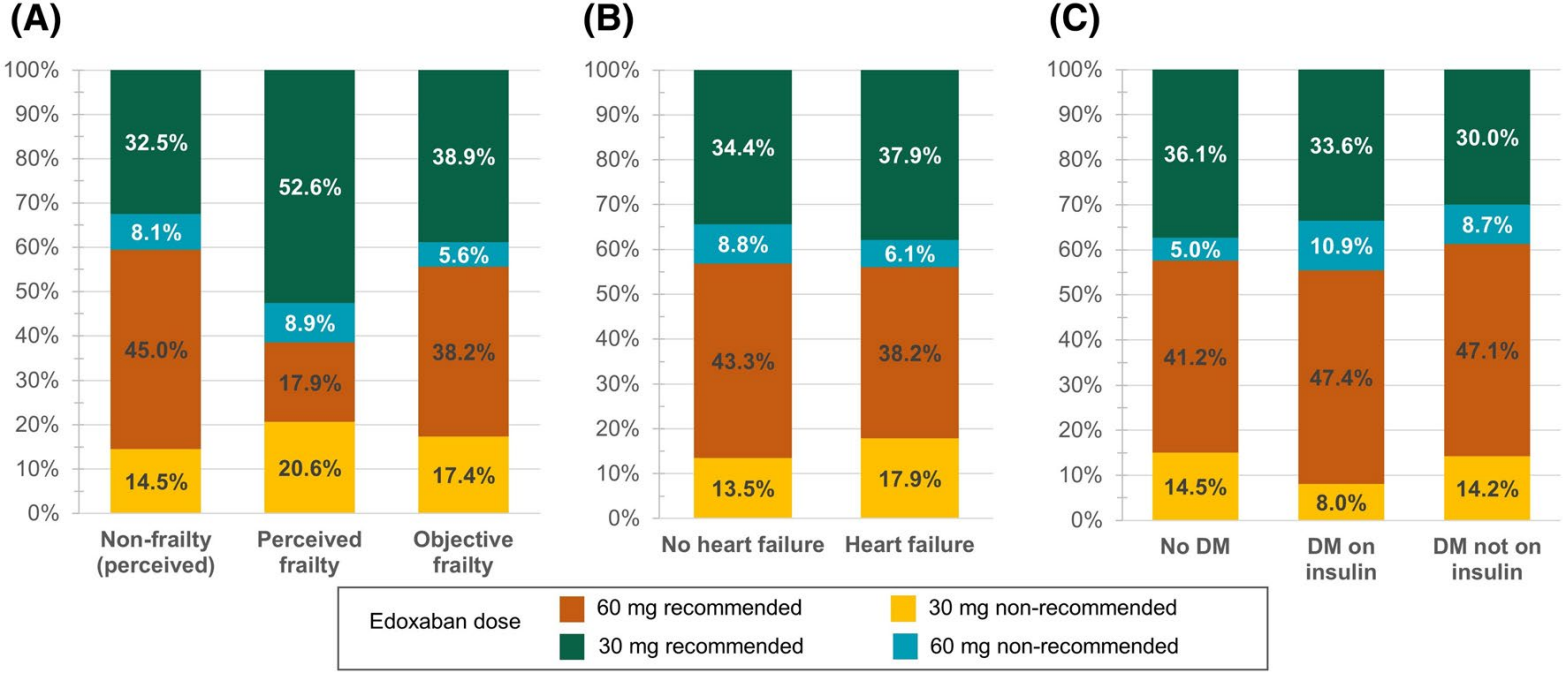
Dose of edoxaban prescribed to (**A**) frailty, (**B**) heart failure and (**C**) diabetes mellitus as label recommendation. DM, Diabetes mellitus.

Compared with the 60 mg recommended dose, patients receiving 30 mg non-recommended doses were older (mean age: 71.3 vs. 66.9 years; 75–84 years: 33.1% vs. 18.4%; ≥ 85 years: 5.7% vs. 1.0%), with higher degree of renal impairment (CrCl: 76.6 vs. 85.7 mL/min; Stage G3: 57.8% vs. 39.4%), CHA_2_DS_2_-VASc (3.04 vs. 2.59) and HAS-BLED scores (1.76 vs. 1.40; Table 2; Fig. 2C–E). Fewer male patients constituted the non-recommended 30 mg group in contrast to the recommended 60 mg prescription (64.4% vs. 71.2%). The 30 mg non-recommendation dose group more often had a history of ischaemic stroke (9.8% vs. 7.0%), previous or concomitant antiplatelet treatment (22.3% vs. 14.7%) and lower VKA use (16.0% vs. 19.5%) compared with the 60 mg recommended dose group (Figure S2 and Fig. 2B). Notably, the proportion of the non-recommended 30 mg treatment was higher in patients with both perceived frailty (20.6%) and objective frailty (17.4%) than those with no frailty (14.5%; Fig. 3A). Patients with history of HF were more frequently treated with non-recommended 30 mg than those with no HF (17.9% vs. 13.5%; Fig. 3B). A percentage of 27.6% patients prescribed with 30 mg non-recommended dose met the exclusion criteria in the previous pivotal randomized controlled trial (RCT) of NOACs, with 34 (5.7%) aged ≥ 85 years, 7 (1.2%) had a history of bleeding from critical area/organ or GI bleeding, 9 (1.5%) were continuously using nonsteroidal anti-inflammatory drugs (NSAIDs), and 134 (22.3%) currently use antiplatelets (Tables S2).

**Table 2:** Baseline characteristics stratified as per label dose recommendation.

	Patients with the label recommendation for 60 mg [N = 2400] (56.7%)		Patients with the label recommendation for 30 mg [N = 1832] (43.3%)	
	60 mg recommended dose [N = 1799] (42.5%)	30 mg non-recommended dose [N = 601] (14.2%)	30 mg recommended dose [N = 1479] (34.9%)	60 mg non-recommended dose [N = 353] (8.3%)
Male, n (%)	1281 (71.2%)	387 (64.4%)	504 (34.1%)	169 (47.9%)
Age (years), mean (SD)	66.9 (8.9)	71.3 (9.1)	74.2 (8.8)	71.5 (8.8)
By age sub-groups, n (%)				
< 65 years	593 (33.0%)	117 (19.5%)	183 (12.4%)	66 (18.7%)
65–74 years	857 (47.6%)	251 (41.8%)	512 (34.6%)	151 (42.8%)
75–84 years	331 (18.4%)	199 (33.1%)	646 (43.7%)	115 (32.6%)
≥ 85 years	18 (1.0%)	34 (5.7%)	138 (9.3%)	21 (5.9%)
Body weight (kg), mean (SD)	75.4 (10.4)	73.0 (8.6)	56.1 (7.9)	60.3 (6.9)
BMI (kg/m^2^), mean (SD)	26.8 (3.3)	26.4 (3.3)	22.1 (2.8)	23.1 (2.6)
SBP (mmHg), mean (SD)	126.6 (15.3)	127.0 (15.8)	124.8 (16.2)	126.3 (15.5)
DBP (mmHg), mean (SD)	77.9 (10.4)	77.3 (10.7)	74.3 (10.3)	76.1 (10.6)
(current) Smokers, n (%)	276 (15.3%)	68 (11.3%)	62 (4.2%)	26 (7.4%)
No alcohol abuse, n (%)	1550 (86.2%)	544 (90.5%)	1338 (90.5%)	303 (85.8%)
(calc) eCrCl (mL/min), mean (SD)	85.7 (25.8)	76.6 (22.6)	51.4 (19.6)	57.5 (19.4)
(calc) eGFR (mL/min/1.73 m^2^), mean (SD)	67.1 (18.1)	61.4 (17.4)	47.7 (18.5)	51.4 (18.2)
(calc) CHA_2_DS_2_-VASc Score, mean (SD)	2.6 (1.4)	3.0 (1.4)	3.4 (1.4)	3.1 (1.3)
(calc) HAS-BLED Score, mean (SD)	1.4 (1.0)	1.8 (0.9)	2.0 (0.8)	1.8 (0.9)
Perceived frailty, n (%)	52 (2.9%)	60 (10.0%)	153 (10.3%)	26 (7.4%)
Objective frailty, n (%)	55 (3.1%)	25 (4.2%)	56 (3.8%)	8 (2.3%)
LVEF < 40%, n (%)	64 (3.6%)	24 (4.0%)	58 (3.9%)	8 (2.3%)
Type of AF, n (%)				
Paroxysmal	667 (37.1%)	227 (37.8%)	570 (38.5%)	122 (34.6%)
Persistent	588 (32.7%)	205 (34.1%)	480 (32.5%)	111 (31.4%)
Long-standing persistent	303 (16.8%)	88 (14.6%)	231 (15.6%)	70 (19.8%)
Permanent	237 (13.2%)	78 (13.0%)	194 (13.1%)	48 (13.6%)
Time since first AF diagnosis (months), mean (SD)	55.2 (70.8)	59.0 (76.8)	50.4 (66.4)	55.1 (76.4)

AF, Atrial fibrillation; BMI, Body mass index; Calc, calculated; CrCl, creatinine clearance; DBP, diastolic blood pressure; eGFR, estimated glomerular filtration rate; LVEF, left ventricular ejection fraction; SBP, systolic blood pressure; SD, standard deviation.

Patients who were prescribed the non-recommended 60 mg had a lower mean age (71.5 vs. 74.2 years; 75–84 years: 32.6% vs. 43.7%; ≥ 85 years: 5.9% vs. 9.3%), better renal function (CrCl: 57.5 vs. 51.4 mL/min; Stage G2: 24.2% vs. 18.9%), and lower CHA_2_DS_2_-VASc (3.1 vs. 3.4) and HAS-BLED scores (1.8 vs. 2.0) than those receiving the recommended 30 mg dose (Table 2; Fig. 2C–E). Patients with the 60 mg non-recommended dose had longer times from first AF diagnosis to edoxaban treatment and a larger percentage had a prior TIA (3.1% vs. 1.9%) than those with the 30 mg recommended dose (Table 2; Figure S2). Furthermore, patients with diabetes were likely to receive non-recommended 60 mg dose regardless of insulin treatment (DM on insulin: 10.9%; DM not on insulin: 8.7%; no DM: 5.0%; Fig. 3C).

### Parameters associated with edoxaban dosing as label recommendation

To better understand the factors associated with edoxaban dosing prescription patterns by physicians, we analysed the clinical characteristics of enrolled patients with AF according to the label recommendation criteria using stepwise logistic regression models (Tables S3–S5).

**Figure 4:**
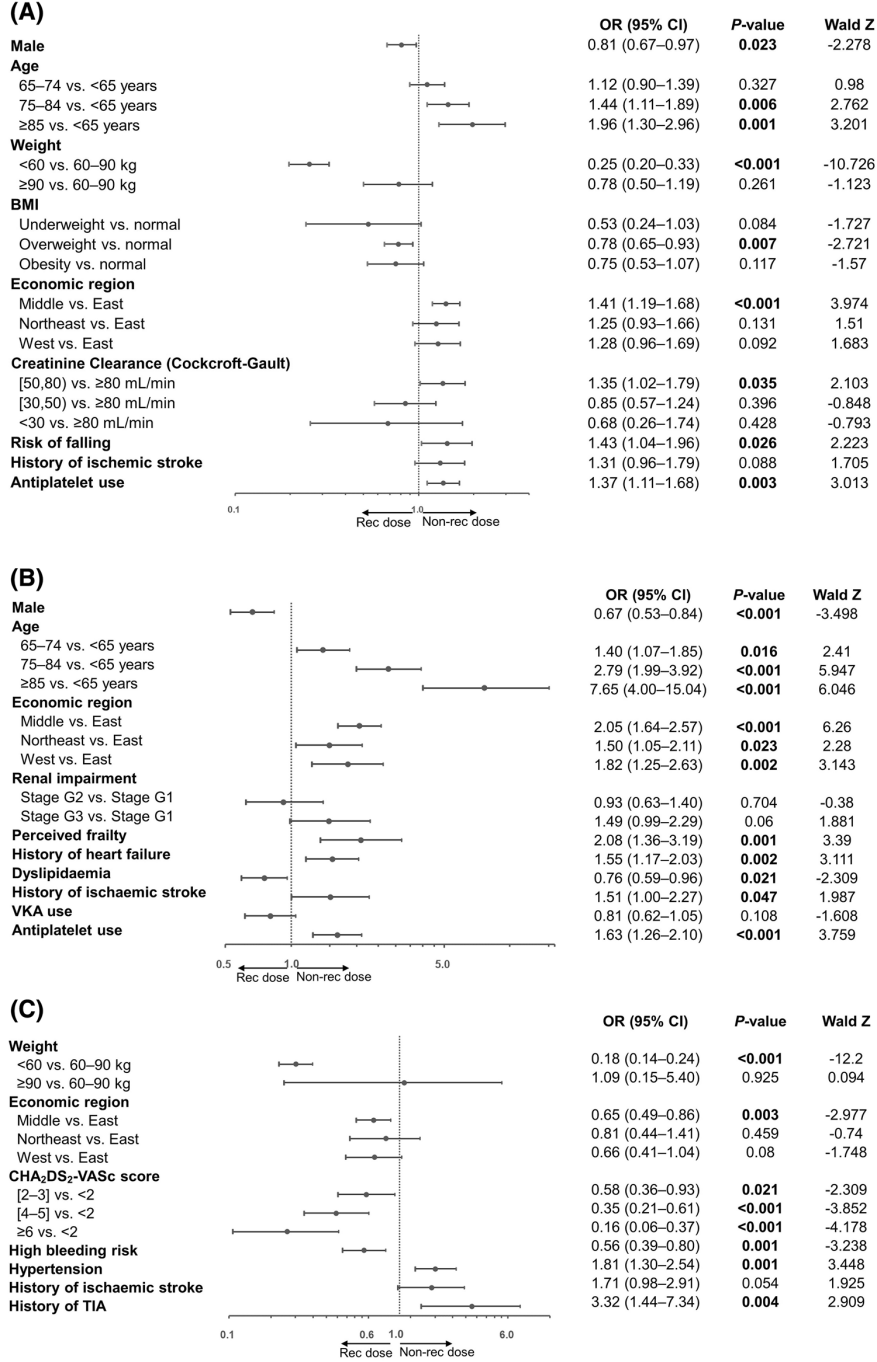
Factors associated not in line with edoxaban label recommendation for (**A**) all doses, (**B**) 60 mg and (**C**) 30 mg in AF patients. BMI categories: normal 18.5–25, underweight < 18.5, overweight 25–30, obesity ≥ 30. Renal impairment stages: Stage G1 eGFR ≥ 90 mL/min/1.73 m^2^, Stage G2 eGFR 60–90 mL/min/1.73 m^2^, Stage G3 eGFR 30–60 mL/min/1.73 m^2^, Stage G4 eGFR 15–30 mL/min/1.73 m^2^, Stage G5 eGFR < 15 mL/min/1.73 m^2^. High–bleeding risk: based on one of the following: creatinine clearance (CrCl) < 50 mL/min, history of bleeding, HAS–BLED > 3. AF, Atrial fibrillation; BMI, Body mass index; CrCl, creatinine clearance; eGFR, estimated glomerular filtration rate; OR, Odds ratio; TIA, transient ischaemic attack; VKA, vitamin K antagonist.

Older age (≥ 85 years: OR 1.96, 95% CI 1.30–2.96, *P* = 0.001; 75–84 years: OR 1.44, 95% CI 1.11–1.89, *P* = 0.006) was the most relevant factor of non-recommended dosing regardless of 60 mg or 30 mg, followed by the risk of fall perceived by physician (OR 1.43, 95% CI 1.04–1.96, *P* = 0.026), middle economic region (OR 1.41, 95% CI 1.19–1.68, *P* < 0.001), antiplatelet use (OR 1.37, 95% CI 1.11–1.68, *P* = 0.003), and CrCl 50–80 mL/min (OR 1.35, 95% CI 1.02–1.79, *P* = 0.035; Fig. 4A). Lower weight (< 60 kg: OR 0.25, 95% CI 0.20–0.33, *P* < 0.001), overweight categorised by BMI (OR 0.78, 95% CI 0.65–0.93, *P* = 0.007) and male gender (OR 0.81, 95% CI 0.67–0.97, *P* = 0.023) were associated with recommended dosing prescription.

All older age groups (≥ 85 years: OR 7.65, 95% CI 4.00–15.04, *P* < 0.001; 75–84 years: OR 2.79, 95% CI 1.99–3.92, *P* < 0.001; and 65–74 years: OR 1.40, 95% CI 1.07–1.85, *P* = 0.016) were strongly associated with the 30 mg non-recommended dose, followed by perceived frailty (OR 2.08, 95% CI 1.36–3.19, *P* = 0.001); Middle economic regions (OR 2.05, 95% CI 1.64–2.57, *P* < 0.001), West (OR 1.82, 95% CI 1.25–2.63, *P* = 0.002), Northeast (OR 1.50, 95% CI 1.05–2.11, *P* = 0.023); antiplatelet use (OR 1.63, 95% CI 1.26–2.10, *P* < 0.001); history of HF (OR 1.55, 95% CI 1.17–2.03, *P* = 0.002); and history of ischaemic stroke (OR 1.51, 95% CI 1.00–2.27, *P* = 0.047; Fig. 4B). Male gender (OR 0.67, 95% CI 0.53–0.84, *P* < 0.001) and dyslipidaemia (OR 0.76, 95% CI 0.59–0.96, *P* = 0.021) were the factors related to the 60 mg recommended dose.

History of TIA (OR 3.32, 95% CI 1.44–7.34, *P* = 0.004) and hypertension (OR 1.81, 95% CI 1.30–2.54, *P* = 0.001) were two strong relevant factors associated with the 60 mg non-recommended dose (Fig. 4C). For 30 mg recommended dose, lower weight (**< **60 kg: OR 0.18, 95% CI 0.14–0.24, *P* < 0.001), higher CHA_2_DS_2_-VASc score (≥ 6: OR 0.16, 95% CI 0.06–0.37, *P* < 0.001; 4–5: OR 0.35, 95% CI 0.21–0.61, *P* < 0.001; 2–3: OR 0.58, 95% CI 0.36–0.93, *P* = 0.021), high bleeding risk (OR 0.56, 95% CI 0.39–0.80, *P* = 0.001) and Middle region (OR 0.65, 95% CI 0.49–0.86, *P* = 0.003) were identified as the associated factors.

## Discussion

This report of baseline characteristics provides the first real-world data throughout Mainland of China of patients routinely treated with edoxaban as the largest, prospective, observational study on a single NOAC. Similar to the ETNA-AF study from other countries/regions, the results indicate patients were enrolled from all age groups, mainly older with mild renal impairment, and were characterised by moderate risk indicated by CHA_2_DS_2_-VASc score, history of CV diseases, stroke, bleeding events and concomitant use of antiplatelet agents, thereby complementing the data of the Phase III ENGAGE AF-TIMI 48 trial^10,28^.

Due to ethnic differences, patients with AF from East Asia, China, presented with distinct demographics, which will be translated into prescription patterns of NOACs in clinical practice. The ETNA-AF-China study is consistent with the XANAP study, regarding the characteristics of enrolled patients, such as the age, BMI, and CV comorbidities including hypertension and DM^29^. The average age of 70.2 years of Chinese patients treated with edoxaban is relatively younger compared with 73.3 years of those with rivaroxaban and 71.9 years with dabigatran in the Hong Kong Atrial Fibrillation Project, and older than the 67.2 years of patients from Mainland of China in the GLORIA-AF registry Phase II (post-approval of dabigatran)^30,31^.

In the ETNA-AF-China study, the mean calculated CHA_2_DS_2_-VASc (2.9) and the HAS-BLED scores (1.6) were lower than in other real-world observational or registry studies of NOACs for Asian patients (3.7 and ~ 2.0)^29,30^. This could be explained by the relatively lower rate of history of ischaemic stroke, TIA, and major bleeding events.

A recent population-based study demonstrated the common treatment changes of OAC in real-world practice^32^. Unlike the XANAP study and the GLORIA-AF registry, very few patients in the ETNA-AF-China study previously used VKA, whereas most of them switched from other NOACs^29,31^. As for Chinese AF patients, the still low use rate of OAC (about 30% of those with CHA_2_DS_2_-VASc score ≥ 1) and very low NOAC use (~ 0.5%) constituted the routine antithrombotic treatment, although improved over time^5,33,34^. The persistence rate of NOACs decreased to 81.3% at 3-year treatment among Chinese patients with AF^35^. Hence, the changes of previous OAC use in the ETNA-AF-China study could also be signified by the considerate long AF diagnosis duration (mean: 54.8 months, Q1–Q3: 4.3–76.5) and a large proportion of persistent (49.5%) AF.

### Prescription patterns with recommended and non-recommended doses

In real-world practice, a total of 45.9% Chinese patients initially received the reduced dose of 30 mg, which is slightly lower than 50.4% in ETNA-AF-Japan but higher than the 23.4% proportion of patients in ETNA-AF-Europe^36,37^. For distribution of dose reduction criteria, ETNA-AF-China revealed that in evaluable patients who met the dose reduction criteria (including recommended and non-recommended doses), 81.4% (*n* = 1491/1832) had a body weight of ≤ 60 kg, 45.9% (*n* = 841/1832) presented a CrCl value of ≤ 50 mL/min, and 0.9% (*n* = 16/1832) were categorised with concomitant use of the label-listed P-gp inhibitor. Together with previous findings, these data show that edoxaban dosing in Asian patients was reduced more on body weight (Japan: 87.5%; Europe: 45.2%), while a larger percentage of patients in Europe received a reduced dose with low CrCl values (Japan: 49.6%; Europe: 84.9%).

There was a good overall label adherence rate of 77.5% with edoxaban routine treatment. In ETNA-AF-China, non-recommended doses were prescribed in 22.5% of the patients, which matches the off-label dosing of NOACs in Europe (22%) but is lower than in Asia (32%) reported by an epidemiological meta-analysis, and greatly below the percentage of the non-recommended dose (> 80%) in the only 65 Chinese patient subgroup of the GARFIELD-AF study^7,21^. We found that 14.2% of patients received edoxaban non-recommended 30 mg instead of the recommended 60 mg called ‘underdose’, and 8.3% of patients received non-recommended 60 mg instead of the recommended 30 mg called ‘overdose’. Of note, the underdose rate of edoxaban in ETNA-AF-China was consistent with previous reported 12.6% in ETNA-AF-Japan, 19.1% in South Korean and Taiwanese patients, with the low overdose rates (2.3%, 10.1%)^56^. Our findings provide the overall prescription pattern of edoxaban to Chinese patients in real-world practice, complementing the data from global ETNA-AF programme.

### Regional diversity of dosing recommendation

In ETNA-AF-China, the prevalence of dosing recommendations showed regional differences. The East region had the lowest percentage of non-recommended 30 mg (10.8%), compared to the West (16.8%), Middle (17.5%), and Northeast region (18.6%). According to the National Bureau of Statistics of China, the East region which accounted for 40% population contributed more than half of the Gross Domestic Product (GDP) as ~ 50-trillion-yuan in 2020, the Middle region contributing similar to that of West (~ 20-trillion-yuan), followed by the Northeast region (~ 5-trillion-yuan)^38^. The total number of tertiary and secondary hospitals (n = 5114), including certified stroke centres (≥ 10%) and registered medical doctors (3.6 million) was relatively high in the East and low in the Middle region (n = 4306, 2.3 million), while West region accounted for 4703 hospitals and 2.0 million registered doctors as obtained from the National Health Commision of China^39^. This high economic level and medical environment associated with the Healthcare Access and Quality (HAQ) index may explain the low probability of underdose NOAC use in East China. Further, we found that the majority of patients in the Northeast region were more likely to receive the recommended 60 mg, while a lowest percentage received non-recommended 60 mg. Epidemiological studies reported the geographical variance in the burden of stroke in China with the Northeastern region having the highest prevalence and incidence of stroke, the highest number of admitted strokes cases and the second highest mortality rate^3,6^. Considering the benefit of reduction in stroke/SEE events with 60 mg vs 30 mg edoxaban, physicians can carefully prescribe a high dose with label recommendation.

### Characteristics and associated factors of NOACs dosing as label recommendation in AF patients

Adherence to OAC is influenced by patient preference, physician’s clinical choice, and the healthcare system^40^. Here, we identified several factors associated with non-recommended dosing, either for both doses or separately for 60 mg or 30 mg, in anticoagulated patients with AF.

Advanced age was a very strongly associated factor of both non-recommended doses and the 30 mg non-recommended low dose. Our findings were in line with the previous studies, that advanced age as a category variable (≥ 75 years, ≥ 65 years) was a strong predictor of non-recommended low dosing of NOACs and as a continuous variable was independently associated with a non-recommended dose routinely used in rivaroxaban^20,21,41^. In real-world setting of Asian countries, low dose of edoxaban was commonly prescribed to older patients (age ≥ 75 years), 87.5% in the ETNA-AF-Japan study, 82.5% in the ANAFIE registry^36,42^. More than a leading factor for AF and related stroke, advanced age is associated with high levels of comorbidities and disability or frailty, emphasising the need of comprehensive consideration on the special patient group^43^.

Some exploration on the association of frailty with dose recommendation has been made. The results demonstrated that the risk of falling is an independent factor for non-recommended dosing, and frailty is a strongly associated factor for non-recommended 30 mg. Consistently, more frail patients from the European countries were treated with edoxaban non-recommended doses, largely with the 30 mg non-recommended dose^26^. In a subgroup analysis of the ENGAGE AF-TIMI 48 trial, patients at a higher risk of falling or frailty experienced more events of major bleeding^44,45^. Data from the ANAFIE registry in older Japanese adults with AF add proof to the association between frailty and CV death, all-cause death and major bleeding^46^. Frailty was common in older patients and more likely accompanied with weight loss, risk of deterioration of renal function and presented as sarcopenia, leading to being bedridden or hospitalisation^47^. Patients with elevated frailty or falling risk received a non-recommended lower dose prescription likely in consideration of safety concerns. Since the increased frailty and risk of falling do not outweigh the benefits of NOAC treatment, precautions such as renal function monitoring and reduction of modifiable risk factors of bleeding assessment are advocated by the EHRA^48^.

History of HF was identified as an associated factor of non-recommended 30 mg. The real-world XANTUS study also reported the similar results that CHF was a predictor of non-recommended dosing^20^. Recently, HF was proven to be the prominent risk factor of all-cause death in AF patients and all-cause and CV death in NOAC users during a long term follow-up of 2 to 5.5 years^13,21,49^. This emphasises the importance of patient-centred as well as multidisciplinary comprehensive management including correct, persistent oral anticoagulation for patients coexisting with AF and HF, as the prevalence of HF in China is increasing within 5 years^50^.

In the current study, hypertension was associated with the 60 mg non-recommended dose. It has been recognised that hypertension contributed to the aetiology of AF and AF-related complications, such as stroke, bleeding risk, and HF^3,50^. Previously, hypertension (including treated with aldosterone antagonists) was identified as a predictor for either inappropriate overdose or underdose in the FANTASIIA and CODE-AF registries^41,51^. In ETNA-AF-China, we found that only a few patients (2.6%) had uncontrolled hypertension (systolic blood pressure [SBP] > 160 mmHg); Moreover, the average diastolic blood pressure (DBP) of non-recommended 60 mg was higher than the recommended 30 mg (76.1 vs. 74.3 mmHg). Notably, a sub-analysis of the ENGAGE AF-TIMI 48 trial showed that patients with DBP of 75–85 mmHg have the most significant reduction in major bleeding on edoxaban treatment compared with a DBP 65–75 mmHg, implicating the pronounced safety profile^52^.

We found that history of TIA was the strongest associated factor with non-recommended 60 mg, and the history of ischaemic stroke was associated with non-recommended 30 mg. Similarly, in the XANTUS study, patients taking the non-recommended dose of a NOAC were more likely to have a prior stroke/TIA^20^. In the GARFIELD-AF registry, a higher percentage of patients receiving non-recommended high dosing of NOACs had history of TIA, and more patients receiving non-recommended low dosing experienced previous stroke than those receiving recommended dosing^21^. The history of TIA or ischaemic stroke as strong predictors for recurrent stroke event has been demonstrated^13^. A stratification analysis of 1,141,097 patients with NVAF initiating oral anticoagulants showed that those at the second-high level of CHA_2_DS_2_-VASc score with a thromboembolic event history had a higher recurrent stroke incidence than those at the highest score level but without an event history, highlighting the independent influence by history of stroke^53^. Indeed, physicians may consider the predictive value of history of ischaemic stroke or TIA on the stroke risk assessment when prescribing, because the higher category of CHA_2_DS_2_-VASc score was associated with the mostly correct dose. Furthermore, patients with AF and a history of ischaemic stroke or TIA were at a higher risk of bleeding when receiving NOAC treatment, which indicated the safety profile consideration on 30 mg non-recommended doses for those patients^54,55^.

Female gender, concomitant antiplatelet use and DM were believed as the predictors of a non-recommended dose or low dose^20,21,41^. In line with the reported data, ETNA-AF-China revealed the association of female gender and antiplatelet use to non-recommended 30 mg prescription. Although dose reduction is recommended for edoxaban and rivaroxaban at CrCl ≤ 50 mL/min, patients with a CrCl value near the threshold were often prescribed a non-recommended dose. Our findings reinforced the understanding and attention to this patient group.

### Non-recommended dosing and clinical implications

Although prescription pattern is well established in this study, optimal dosing for patients for the very elderly or high risk of bleeding remains a challenge for physicians. Globally, ‘underdosed’ edoxaban was reported to be associated with a higher risk of all-cause and CV death, while ‘overdosed’ had lower risk of ischaemic stroke without an increased risk of major bleeding^56^. In Asian patients with AF, several real world studies have found that non-recommended low dose NOACs are associated with reduced benefits, such as a higher risk of ischaemic stroke/SEE, and non-recommended high dose NOACs with similar risk of major bleeding^57,58^. In ETNA-AF-China, 435 patients with non-recommended 30 mg edoxaban in the ENGAGE AF-TIMI 48 trial met the eligibility criteria, the prescription of whom should be avoided. However, 166 (27.6%) patients were not included in the pivotal RCTs because they were older than 85 years, had a history of bleeding from critical organ or GI bleeding, concomitant use of NSAIDs, antiplatelets and 60 (10.0%) patients were considered frail. The ELDERCARE-AF trial showed that very low dose of edoxaban (15 mg OD) effectively prevented stroke or SEE, with no significant rising major bleeding risk in very elderly Asian patients with AF and high risk of bleeding^59^. From an Asian perspective, in patients with very advanced age, high bleeding risk or perceived frailty who did not meet the pivotal RCTs conclusion, flexible dosing could be considered based on shared physician judgement. Future studies on the ongoing ETNA-AF-China trial will assess the clinical impact of non-recommended edoxaban dosing for Chinese patients.

### Limitations

Owing to the design of observational study, adherence of medication or receiving the prescribed dose could not be ensured, and treatment duration in different dosing groups were not compared. Approximately 14% (698/5001) of patients could not be characterised as recipients of recommended or non-recommended dosing, mainly due to missing CrCl values, and the values could not be retrieved due to the study’s nature as a registry. Finally, like in all open-label studies, patients were aware about the treatment, and made decision to provide consent, which may have introduced some selection and ascertainment bias.

## Conclusion

Edoxaban treatment in clinical practice in China was seen across all age groups and comorbidities, with a majority of patients being older than 65 years old with AF and having mild-to-moderate renal impairment. The adherence to label recommendation on dosing was high (77.5%), with slight regional variation. Advanced age was identified as the most relevant factor associated with non-recommended doses, with frailty having strongly association for the non-recommended 30 mg, and history of TIA for non-recommended 60 mg. These data demonstrate the baseline characteristics and prescription pattern of edoxaban in routine care in ETNA-AF-China and indicate the need for patient-centred, comorbidity management optimisation for improvement on future outcomes.

### Supplementary Information

Supplementary Information.

## Data Availability

The data underlying this article are available in the article and in its online Supplementary material. Further data underlying this article will be shared from the corresponding author on reasonable request.
