# Supplementary material for: Edoxaban for stroke prevention in atrial fibrillation and factors associated with dosing: patient characteristics from the prospective observational ETNA-AF-China registry
Source: Sci Rep. 2024 Feb 2;14:2778. doi: 10.1038/s41598-024-51776-3 (PMC10837439; doi:10.1038/s41598-024-51776-3)
Supplement: Supplementary file 1 — Supplementary Information. [file 41598_2024_51776_MOESM1_ESM.docx]

**Supplementary data**

**
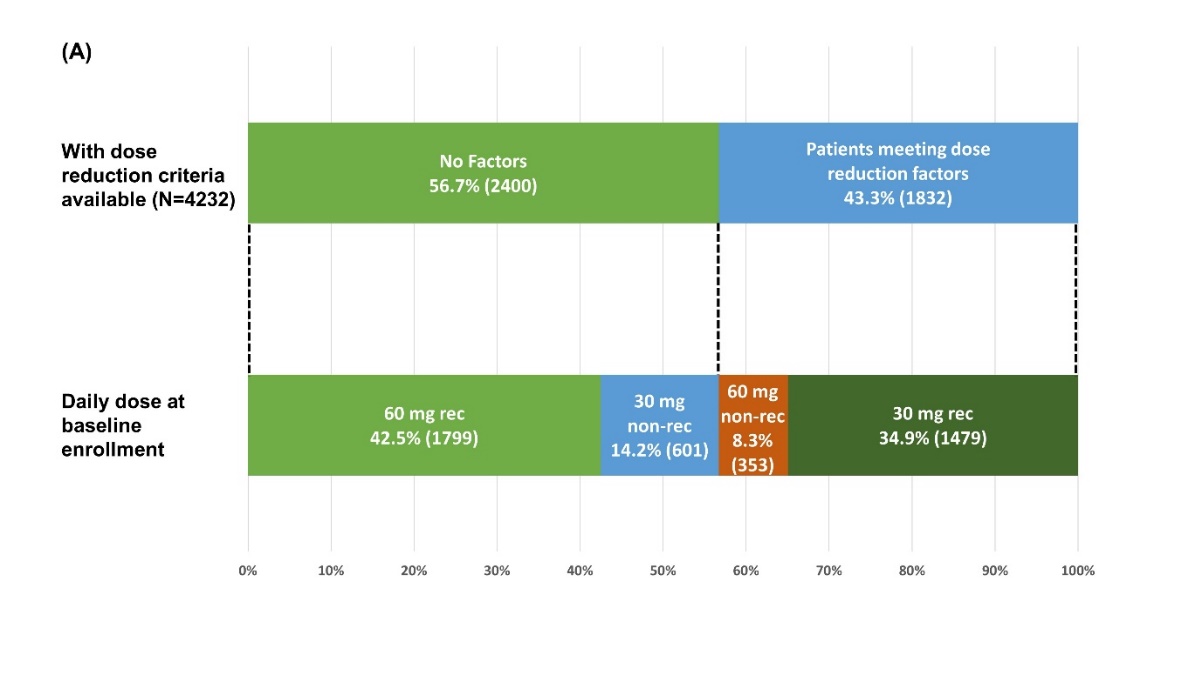
**

**
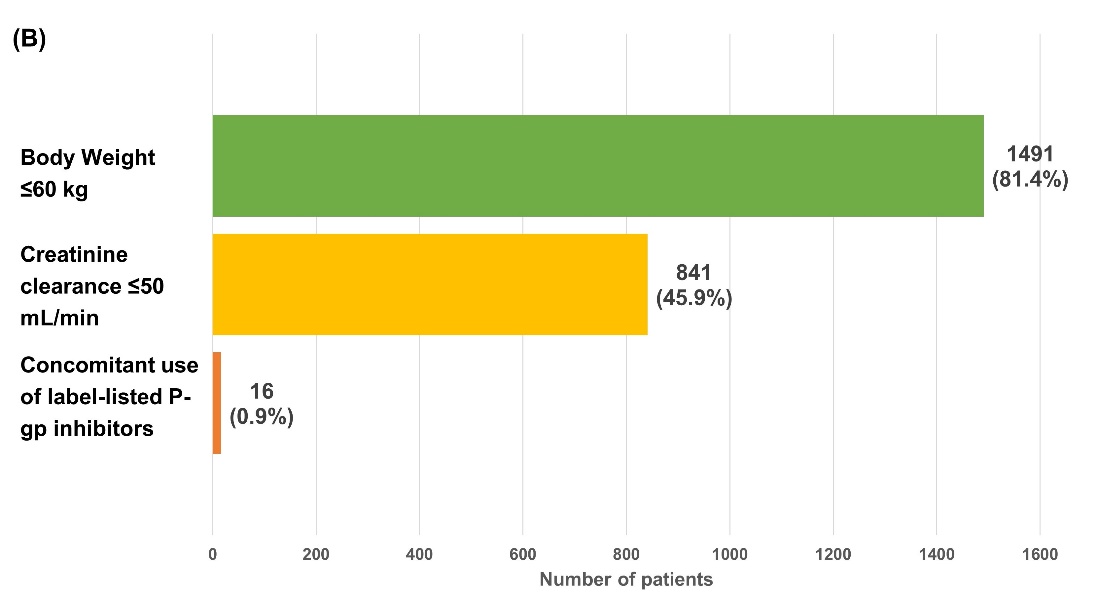
**

**Figure S1** Edoxaban dose recommendations and distribution of dose reduction criteria

Rec, recommended; Non-rec, non-recommended; CrCl, creatinine clearance; P-gp, P-glycoprotein.


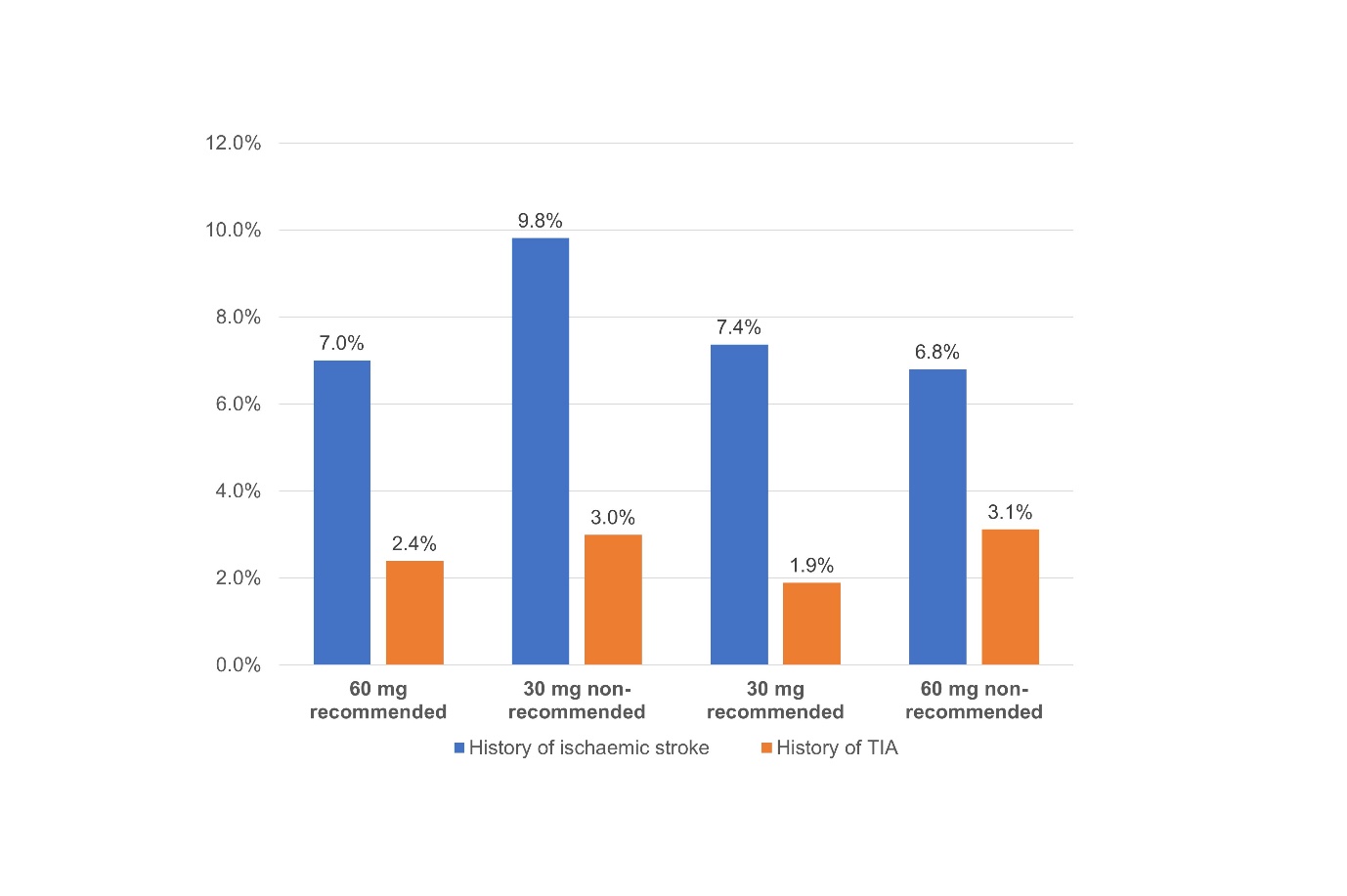


**Figure S2** Distribution of history of ischaemic stroke and TIA on patients categorised by dose recommendation

TIA, Transient ischaemic attack


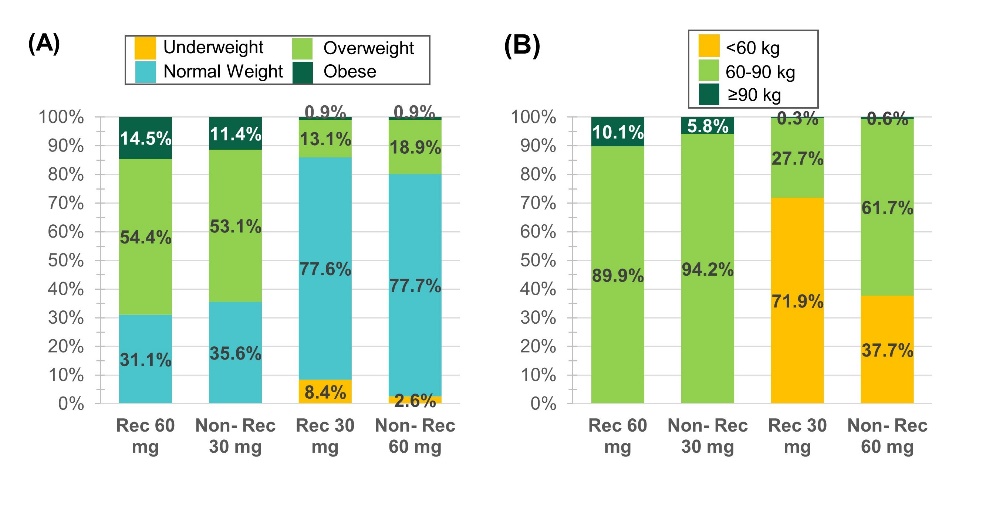


**Figure S3** Distribution of weight and BMI on patients categorised by dose recommendation.

BMI, Body mass index

**Table S1** Investigators and their affiliated centers in ETNA-AF-China

| **ID** | **Site** | **Economic region** | **Province** | **City** | **Investigator** |
| --- | --- | --- | --- | --- | --- |
| 1 | The Second Affiliated Hospital of Shenyang Medical College | Northeast | Liaoning | Shenyang | Yang Yang |
| 2 | Beijing Anzhen Hospital, Capital Medical University | East | Beijing | Beijing | Changsheng Ma |
| 3 | Xiangtan Central Hospital | Middle | Hunan | Xiangtan | Mingxing Wu |
| 4 | The Second Affiliated Hospital of Guangzhou Medical University | East | Guangdong | Guangzhou | Wenchao Qu |
| 5 | Shanxi Cardiovascular Hospital | Middle | Shanxi | Taiyuan | Xuebin Han |
| 6 | Xinxiang Central Hospital | Middle | Henan | Xinxiang | Zhifang Wang |
| 7 | The Fourh Hospital of Changsha | Middle | Hunan | Changsha | Jing Jin |
| 8 | Tsinghua University Affiliated Beijing Tsinghua Changgeng Hospital | East | Beijing | Beijing | Ping Zhang |
| 9 | Qingpu Branch of Zhongshan Hospital Affiliated to Fudan University | East | Shanghai | Shanghai | Zheng Zhang |
| 10 | Guangzhou Panyu Central Hospital | East | Guangdong | Guangzhou | Guoqin Chen |
| 11 | The Second Affiliated Hospital of Nanjing Medical University | East | Jiangsu | Nanjing | Mingzhi Long |
| 12 | The Third Affiliated Hospital of Xinxiang Medical University | Middle | Henan | Xinxiang | Guotian Yin |
| 13 | The Second Hospital of Tianjin Medical University | East | Tianjin | Tianjin | Tong Liu |
| 14 | Affiliated Hospital of Jiangnan University | East | Jiangsu | Wuxi | Xiaoyan Wang |
| 15 | Wuhan Third Hospital | Middle | Hubei | Wuhan | Dongsheng Li |
| 16 | The Central Hospital of Wuhan | Middle | Hubei | Wuhan | Manhua Chen |
| 17 | The First Affiliated Hospital, Sun Yat-Sen University | East | Guangdong | Guangzhou | Yugang Dong |
| 18 | Shanxi Provincial People’s Hospital | Middle | Shanxi | Taiyuan | Chunlin Lai |
| 20 | Jilin Province People’s Hospital | Northeast | Jilin | Changchun | Xuelian Zhang |
| 19 | The Second Affiliated Hospital of Chongqing Medical University | West | Chongqing | Chongqing | Yuehui Yin |
| 21 | The First Hospital of Changsha | Middle | Hunan | Changsha | Heng Qi |
| 22 | Wenzhou Central Hospital | East | Zhejiang | Wenzhou | Xiaojun Ji |
| 23 | Nanjing Jiangning Hospital | East | Jiangsu | Nanjing | Yuqing Zhang |
| 24 | The Fifth People's Hospital of Jinan | East | Shandong | Jinan | Zhisheng Jia |
| 25 | The Fifth Affiliated Hospital of Zhengzhou University | Middle | Henan | Zhengzhou | Xue Liang |
| 26 | Longhua Hospital Shanghai University of Traditional Chinese Medicine | East | Shanghai | Shanghai | Ping Deng |
| 27 | Kaifeng Central Hospital | Middle | Henan | Kaifeng | Jieyun Liu |
| 28 | The First Hospital of Kunming | West | Yunnan | Kunming | Juan Ma/Cangsang Song |
| 29 | Taiyuan Central Hospital of Shanxi Medical University | Middle | Shanxi | Taiyuan | Huifang Feng |
| 30 | The First Affiliated Hospital of Chongqing Medical University | West | Chongqing | Chongqing | Suxin Luo |
| 31 | Sun Yat-sen Memorial hospital, Sun Yat-sen University | East | Guangdong | Guangzhou | Jingfeng Wang |
| 32 | Wuhan Puai Hospital | Middle | Hubei | Wuhan | Lun Li |
| 33 | The Third People's Hospital of Nanning | West | Guangxi | Nanning | Yongqi Xiao |
| 34 | Jiangyin People's Hospital | East | Jiangsu | Wuxi | Junyou Cui |
| 35 | Union Hospital Affiliated With Tongji Medical College of Huazhong University of Science and Technology | Middle | Hubei | Wuhan | Xiang Cheng |
| 36 | The First Affiliated Hospital of Guangzhou Medical University | East | Guangdong | Guangzhou | Zheng Huang |
| 37 | West China Hospital, Sichuan University | West | Sichuan | Chengdu | Jiafu Wei |
| 38 | Shanxi Bethune Hospital | Middle | Shanxi | Taiyuan | Jinfang Cheng |
| 39 | Changzhou No.2 People’s Hospital | East | Jiangsu | Changzhou | Haiyan Li |
| 40 | Suzhou Municipal Hospital | East | Jiangsu | Suzhou | Jun Zhang |
| 41 | The Third Xiangya Hospital of Central South University | Middle | Hunan | Changsha | Weihong Jiang |
| 42 | The First People's Hospital of Nanning | West | Guangxi | Nanning | Jie Liu |
| 43 | Dongfeng General Hospital of Chinese Medicine | Middle | Hubei | Shiyan | Xinwen Min |
| 44 | Peking Union Medical College Hospital | East | Beijing | Beijing | Peng Gao |
| 45 | The Fist Affiliated Hospital of Dalian Medical University | Northeast | Liaoning | Dalian | Lianjun Gao |
| 46 | The First Affiliated Hospital of Wenzhou Medical University | East | Zhejiang | Wenzhou | Zhouqing Huang |
| 47 | Wuxi People's Hospital | East | Jiangsu | Wuxi | Ruxing Wang |
| 48 | Shanghai East Hospital, Tongji University | East | Shanghai | Shanghai | Ying Li |
| 49 | Guangdong Second Provincial General Hospital | East | Guangdong | Guangzhou | Zebin Ye |
| 50 | The Fourth Hopital of Jilin University | Northeast | Jilin | Changchun | Xubo Wang |
| 51 | Jiangxi Provincial People’s Hospital | Middle | Jiangxi | Nanchang | Hengli Lai |
| 52 | Zhejiang Provincial People’s Hospital | East | Zhejiang | Hangzhou | Lihong Wang |
| 53 | Hebei Petro China Center Hospital | East | Hebei | Langfang | Minli Zhang |
| 54 | Shanghai Ninth People’s Hospital - Shanghai Jiaotong University School of Medicine | East | Shanghai | Shanghai | Changqian Wang |
| 55 | Zhejiang Provincial Hospital of Chinese Medinice | East | Zhejiang | Hangzhou | Wei Mao |
| 56 | The First People's Hospital of Kunshan | East | Jiangsu | Suzhou | Xiaohua Chen |
| 57 | The Affiliated Hospital of Xuzhou Medical University | East | Jiangsu | Xuzhou | Zhirong Wang |
| 58 | Aviation General Hospital | East | Beijing | Beijing | Fang Liu |
| 59 | Hangzhou Third Hospital | East | Zhejiang | Hangzhou | Xiaolin Xie |
| 60 | Zhejiang Greentown Cardiovascular Hospital | East | Zhejiang | Hangzhou | Zhenggui Xu |
| 61 | Heping Hospital Affiliated to Changzhi Medical College | Middle | Shanxi | Changzhi | Bin Dong |
| 62 | Tongji Hospital Affiliated to Tongji Medical College of Huazhong University of Science and Technology | Middle | Hubei | Wuhan | Hesong Zeng |
| 63 | Hunan Provincial People’s Hospital | Middle | Hunan | Changsha | Jianqiang Peng |
| 64 | The Second Affiliated Hospital of Zhengzhou University | Middle | Henan | Zhengzhou | Lihua Zhang |
| 65 | Renmin Hospital of Wuhan University - Hubei General Hospital | Middle | Hubei | Wuhan | Qingyan Zhao |
| 66 | The First Affiliated Hospital of Jinan University | East | Guangdong | Guangzhou | Fucheng Liu |
| 67 | Beijing Tongren Hospital | East | Beijing | Beijing | Caixia Guo |
| 68 | Zhejiang Hospital | East | Zhejiang | Hangzhou | Lijiang Tang |
| 69 | Xuzhou Central Hospital | East | Jiangsu | Xuzhou | Xianjin Li |
| 70 | Peking University First Hospital | East | Beijing | Beijing | Jing Zhou |
| 71 | Beijing Chao-Yang Hospital Capital Medical University | East | Beijing | Beijing | Xinchun Yang |
| 72 | Wenzhou People's Hospital | East | Zhejiang | Wenzhou | Xiaoshu Chen |
| 73 | Beijing Miyun District Hospital | East | Beijing | Beijing | Yajuan Liu |
| 74 | China Shenyang Chest Hospital | Northeast | Liaoning | Shenyang | Tiebi tong |
| 75 | The First Hospital of Jiaxing | East | Zhejiang | Jiaxing | Guanmin Tang |
| 76 | Guangzhou First People’s Hospital | East | Guangdong | Guangzhou | Jian Liu |
| 77 | Wenzhou Hospital of Traditional Chinese Medicine | East | Zhejiang | Wenzhou | Meise Lin |
| 78 | The Second Hospital of Jilin Hospital | Northeast | Jilin | Changchun | Bin Liu |
| 79 | Sir Run Run Shaw Hospital - Zhejiang University School of Medicine | East | Zhejiang | Hangzhou | Guosheng Fu |
| 80 | Heilongjiang Provincial Hospital | Northeast | Heilongjiang | Haerbin | Weimin Wang |
| 81 | The General Hospital of Tianjin Medical University | East | Tianjin | Tianjin | Qing Yang |
| 82 | The First Affiliated Hospital of Xinjiang Medical University | West | Xinjiang | Wulumuqi | Baopeng Tang |
| 83 | Chongqing General Hospital | West | Chongqing | Chongqing | Boli Ran |
| 84 | The Second Hospital of Jiaxing | East | Zhejiang | Jiaxing | Jianjiang Xu |
| 85 | Xuanwu Hospital Capital Medical University | East | Beijing | Beijing | Chunlin Yin |
| 86 | Zhujiang Hospital of Southern Medical University | East | Guangdong | Guangzhou | Pingzhen Yang |
| 87 | Beijing Hospital | East | Beijing | Beijing | Fujie Yang |
| 88 | The First Hospital of Lanzhou University | West | Gansu | Lanzhou | Ming Bai |
| 89 | Beijing Chest Hospital, Capital Medical University | East | Beijing | Beijing | Jian Zhang |

**Table S2** Characteristics of patients prescribed with 30 mg non-recommended dose excluded in pivotal RCTs

|  | 30 mg non-recommended dose [N = 601] | |
| --- | --- | --- |
|  | **Patients who excluded in pivotal RCTs**^*^  **[N=166] (27.6%)** | **Patients who were well presented in pivotal RCTs**  **[N =** **435] (72.4%)** |
| Age ≥ 85 years | 34 (5.7%) | 567 (94.3%) |
| CrCl 15–30 mL/min, n (%) | 0 (0%) | 601 (100%) |
| History of bleeding from critical area/organ or GI bleeding | 7(1.2%) | 594 (98.8%) |
| Low body weight (≤ 45 kg), n (%) | 0 (0%) | 601 (100%) |
| Continuous use of NSAIDs | 9 (1.5%) | 592 (98.5%) |
| Current use of antiplatelets | 134 (22.3%) | 467 (77.7%) |

^*^Patients who met one of exclusion criteria, such as age ≥ 85 years, CrCl 15–30 mL/min, history of bleeding from critical area/organ or GI bleeding, body weight ≤ 45 kg, continuous use of NSAIDs, current use of antiplatelets could be categorized in subgroup of those excluded in pivotal RCTs.

RCT, randomized controlled trial; CrCl, creatinine clearance; NSAIDs, nonsteroidal anti-inflammatory drugs.

**Table S3** Factors associated with edoxaban treatment not in line with label recommendation in patients with AF

|  | **Univariable** | | | **Multivariable** | | |
| --- | --- | --- | --- | --- | --- | --- |
|  | **OR (95% CI)** | **P-value** | **Wald Z** | **OR (95% CI)** | **P-value** | **Wald Z** |
| Male | 1.168 (1.01–1.353) | **0.036** | 2.091 | 0.809 (0.674–0.971) | **0.023** | -2.278 |
| Age |  |  |  |  |  |  |
| 65–74 vs. <65 years | 1.245 (1.025–1.517) | **0.028** | 2.196 | 1.116 (0.897–1.39) | 0.327 | 0.98 |
| 75–84 vs. <65 years | 1.363 (1.111–1.675) | **0.003** | 2.957 | 1.448 (1.114–1.885) | **0.006** | 2.762 |
| ≥85 vs. <65 years | 1.495 (1.05–2.105) | **0.023** | 2.271 | 1.964 (1.295–2.962) | **0.001** | 3.201 |
| Weight |  |  |  |  |  |  |
| <60 vs. 60–90 kg | 0.326 (0.267–0.396) | **<0.001** | -11.102 | 0.254 (0.197–0.325) | **<0.001** | -10.726 |
| ≥90 vs. 60–90 kg | 0.511 (0.351–0.725) | **<0.001** | -3.631 | 0.782 (0.503–1.191) | 0.261 | -1.123 |
| BMI |  |  |  |  |  |  |
| Underweight vs. normal | 0.257 (0.121–0.482) | **<0.001** | -3.886 | 0.534 (0.244–1.033) | 0.084 | -1.727 |
| Overweight vs. normal | 1.138 (0.975–1.327) | 0.1 | 1.643 | 0.779 (0.651–0.933) | **0.007** | -2.721 |
| Obesity vs. normal | 0.898 (0.673–1.183) | 0.453 | -0.751 | 0.753 (0.526–1.069) | 0.117 | -1.57 |
| Economic region |  |  |  |  |  |  |
| Middle | 1.338 (1.14–1.57) | **<0.001** | 3.572 | 1.414 (1.192–1.678) | **<0.001** | 3.974 |
| Northeast | 1.253 (0.953–1.634) | 0.1 | 1.643 | 1.25 (0.932–1.664) | 0.131 | 1.51 |
| West | 1.324 (1.013–1.715) | **0.037** | 2.09 | 1.275 (0.957–1.687) | 0.092 | 1.683 |
| Smoking |  |  |  |  |  |  |
| Current vs. never | 0.978 (0.763–1.244) | 0.859 | -0.177 |  |  |  |
| Formerly vs. never | 1.182 (0.947–1.467) | 0.134 | 1.497 |  |  |  |
| Alcohol abuse | 0.895 (0.6–1.299) | 0.572 | -0.566 |  |  |  |
| Creatinine Clearance (Cockcroft-Gault) |  |  |  |  |  |  |
| [50,80) vs. ≥80 mL/min | 1.616 (1.362–1.921) | **<0.001** | 5.462 | 1.354 (1.019–1.793) | **0.035** | 2.103 |
| [30,50) vs. ≥80 mL/min | 0.887 (0.697–1.124) | 0.323 | -0.988 | 0.846 (0.574–1.243) | 0.396 | -0.848 |
| <30 vs. ≥80 mL/min | 0.38 (0.19–0.683) | **0.003** | -3.002 | 0.681 (0.258–1.739) | 0.428 | -0.793 |
| Renal impairment |  |  |  |  |  |  |
| Stage G2 vs. Stage G1 | 1.053 (0.759–1.484) | 0.764 | 0.3 | 0.896 (0.628–1.296) | 0.552 | -0.595 |
| Stage G3 vs. Stage G1 | 1.493 (1.089–2.082) | **0.015** | 2.426 | 1.166 (0.751–1.827) | 0.499 | 0.676 |
| ≥Stage G4 vs. Stage G1 | 0.518 (0.294–0.884) | **0.019** | -2.355 | 0.669 (0.293–1.466) | 0.325 | -0.983 |
| CHA_2_DS_2_-VASc score |  |  |  |  |  |  |
| [2–3] vs. <2 | 1.22 (0.98–1.529) | 0.079 | 1.754 |  |  |  |
| [4–5] vs. <2 | 1.197 (0.944–1.525) | 0.14 | 1.475 |  |  |  |
| ≥6 vs. <2 | 1.177 (0.788–1.734) | 0.416 | 0.813 |  |  |  |
| HAS-BLED score |  |  |  |  |  |  |
| 2 vs. <2 | 1.283 (1.079–1.527) | 0.005 | 2.816 |  |  |  |
| ≥3 vs. <2 | 1.592 (1.261–2.004) | <0.001 | 3.934 |  |  |  |
| High risk of stroke or bleeding^a^ | 0.993 (0.854–1.154) | 0.931 | -0.086 | 0.844 (0.682–1.043) | 0.118 | -1.562 |
| High bleeding risk^b^ | 0.727 (0.607–0.868) | **<0.001** | -3.496 |  |  |  |
| Frailty | 1.441 (1.101–1.872) | **0.007** | 2.702 |  |  |  |
| Risk of fall | 1.343 (0.388–6.177) | 0.665 | 0.433 | 1.432 (1.04–1.96) | **0.026** | 2.223 |
| LVEF <40 % | 0.843 (0.556–1.244) | 0.404 | -0.834 |  |  |  |
| Hypertension | 1.104 (0.937–1.303) | 0.242 | 1.169 |  |  |  |
| History of heart failure | 1.1 (0.902–1.335) | 0.342 | 0.95 |  |  |  |
| Coronary heart disease | 1.09 (0.943–1.26) | 0.245 | 1.163 |  |  |  |
| Valvular disease | 1.048 (0.762–1.421) | 0.767 | 0.296 |  |  |  |
| Peripheral artery disease | 0.613 (0.352–1.005) | 0.066 | -1.839 |  |  |  |
| Diabetes mellitus |  |  |  |  |  |  |
| DM not on insulin vs. No DM | 1.011 (0.84–1.212) | 0.909 | 0.115 |  |  |  |
| DM on insulin vs. No DM | 0.795 (0.505–1.209) | 0.303 | -1.03 |  |  |  |
| COPD | 1.391 (1.02–1.874) | **0.033** | 2.128 |  |  |  |
| Dyslipidaemia | 0.892 (0.752–1.056) | 0.189 | -1.313 |  |  |  |
| Chronic hepatic disease | 0.943 (0.655–1.328) | 0.743 | -0.328 | 0.987 (0.675–1.414) | 0.943 | -0.072 |
| History of stroke | 1.112 (0.871–1.408) | 0.386 | 0.867 |  |  |  |
| History of ischaemic stroke | 1.234 (0.946–1.596) | 0.115 | 1.576 | 1.312 (0.957–1.787) | 0.088 | 1.705 |
| History of TIA | 1.416 (0.901–2.171) | 0.12 | 1.557 |  |  |  |
| History of ischaemic stroke or TIA | 1.251 (0.985–1.577) | 0.063 | 1.862 |  |  |  |
| Previous ICH | 0.571 (0.167–1.482) | 0.3 | -1.036 |  |  |  |
| Previous major bleeding | 0.526 (0.199–1.155) | 0.144 | -1.461 | 0.544 (0.201–1.243) | 0.183 | -1.332 |
| Previous major or CRNM bleeding | 0.622 (0.27–1.253) | 0.218 | -1.232 |  |  |  |
| VKA use | 0.821 (0.677–0.992) | **0.043** | -2.023 | 0.866 (0.707–1.055) | 0.157 | -1.414 |
| Other NOACs use | 0.963 (0.833–1.113) | 0.613 | -0.506 |  |  |  |
| Antiarrhythmics use | 0.998 (0.863–1.153) | 0.975 | -0.032 |  |  |  |
| Antiplatelet use | 1.351 (1.118–1.628) | **0.002** | 3.137 | 1.368 (1.114-1.675) | **0.003** | 3.013 |
| NASAIDs use | 1.269 (0.674–2.369) | 0.455 | 0.747 |  |  |  |
| P-gps use (edoxaban label listed) | 4.419 (1.546–12.658) | **0.005** | 2.815 | 5.117 (1.633–16.29) | **0.005** | 2.817 |

^a^High risk of stroke or bleeding was considered based on one of the following: prior stroke, prior major bleeding, prior ICH or (calculated) CHA2DS2-VASc ≥4. ^b^High bleeding risk was considered based on one of the following: creatinine clearance (CrCl) <50 mL/min, history of bleeding, HAS-BLED >3.

AF, Atrial fibrillation; BMI, Body mass index; Calc, calculated; CHF, Congestive heart failure; COPD, chronic obstructive pulmonary disease; CRNM, clinically relevant non–major; DM, Diabetes mellitus; ICH, intracranial haemorrhage; LVEF, left ventricular ejection fraction; NSAID, Non–steroidal anti–inflammatory drugs; OD, Odds Ratio; P-gp, P-glycoprotein; TIA, transient ischaemic attack; VKA, vitamin K antagonist; SD, standard deviation.

**Table S4** Factors associated edoxaban treatment not in line with 60 mg package insert recommendation in AF patients

|  | **Univariable** | | | **Multivariable** | | |
| --- | --- | --- | --- | --- | --- | --- |
|  | **OR (95% CI)** | **P-value** | **Wald Z** | **OR (95% CI)** | **P-value** | **Wald Z** |
| Male | 0.731 (0.602–0.89) | **0.002** | -3.135 | 0.666 (0.53–0.837) | **<0.001** | -3.498 |
| Age |  |  |  |  |  |  |
| 65–74 vs. <65 years | 1.484 (1.167–1.898) | **0.001** | 3.185 | 1.4 (1.067–1.845) | **0.016** | 2.41 |
| 75–84 vs. <65 years | 3.047 (2.342–3.98) | **<0.001** | 8.241 | 2.789 (1.992–3.918) | **<0.001** | 5.947 |
| ≥85 vs. <65 years | 9.574 (5.293–17.864) | **<0.001** | 7.321 | 7.654 (3.996–15.04) | **<0.001** | 6.046 |
| Weight |  |  |  |  |  |  |
| ≥90 vs. 60–90 kg | 0.549 (0.372–0.789) | **0.002** | -3.137 |  |  |  |
| BMI |  |  |  |  |  |  |
| Overweight vs. normal | 0.852 (0.695–1.045) | 0.122 | -1.546 |  |  |  |
| Obesity vs. normal | 0.685 (0.499–0.932) | **0.017** | -2.377 |  |  |  |
| Economic region |  |  |  |  |  |  |
| Middle | 2.092 (1.698–2.578) | **<0.001** | 6.931 | 2.05 (1.638–2.569) | **<0.001** | 6.26 |
| Northeast | 1.52 (1.099–2.084) | **0.01** | 2.568 | 1.497 (1.053–2.109) | **0.023** | 2.28 |
| West | 2.301 (1.622–3.238) | **<0.001** | 4.732 | 1.82 (1.247–2.633) | **0.002** | 3.143 |
| Smoking |  |  |  |  |  |  |
| Current vs. never | 0.69 (0.514–0.916) | **0.012** | -2.519 |  |  |  |
| Formerly vs. never | 0.958 (0.736–1.238) | 0.745 | -0.325 |  |  |  |
| Alcohol abuse | 0.617 (0.381–0.958) | **0.039** | -2.06 |  |  |  |
| Creatinine Clearance (Cockcroft-Gault) |  |  |  |  |  |  |
| [50,80) vs. ≥80 mL/min | 1.998 (1.652–2.423) | **<0.001** | 7.08 |  |  |  |
| Renal impairment |  |  |  |  |  |  |
| Stage G2 vs. Stage G1 | 1.095 (0.758–1.614) | 0.637 | 0.471 | 0.925 (0.624–1.396) | 0.704 | -0.38 |
| Stage G3 vs. Stage G1 | 2.27 (1.585–3.323) | **<0.001** | 4.353 | 1.494 (0.991–2.292) | 0.06 | 1.881 |
| CHA_2_DS_2_-VASc score |  |  |  |  |  |  |
| [2–3] vs. <2 | 1.52 (1.163–2.005) | **0.003** | 3.019 |  |  |  |
| [4–5] vs. <2 | 2.146 (1.592–2.911) | **<0.001** | 4.963 |  |  |  |
| ≥6 vs. <2 | 3.758 (2.206–6.376) | **<0.001** | 4.905 |  |  |  |
| HAS-BLED score |  |  |  |  |  |  |
| 2 vs. <2 | 2.071 (1.669–2.574) | **<0.001** | 6.592 |  |  |  |
| ≥3 vs. <2 | 2.767 (2.058–3.713) | **<0.001** | 6.769 |  |  |  |
| High risk of stroke or bleeding | 1.566 (1.285–1.906) | **<0.001** | 4.46 | 0.748 (0.56–0.995) | **0.048** | -1.981 |
| High bleeding risk | 1.378 (0.919–2.03) | 0.111 | 1.592 |  |  |  |
| Frailty | 3.583 (2.438–5.283) | **<0.001** | 6.484 | 2.083 (1.363–3.189) | **0.001** | 3.39 |
| Risk of fall | 3.75 (0.462–77.253) | 0.26 | 1.128 |  |  |  |
| LVEF <40 % | 1.082 (0.654–1.735) | 0.751 | 0.318 |  |  |  |
| Hypertension | 0.91 (0.737–1.127) | 0.381 | -0.875 |  |  |  |
| History of heart failure | 1.498 (1.173–1.904) | **0.001** | 3.271 | 1.545 (1.172–2.03) | **0.002** | 3.111 |
| Coronary heart disease | 1.339 (1.112–1.614) | **0.002** | 3.076 |  |  |  |
| Valvular disease | 1.305 (0.848–1.966) | 0.213 | 1.246 |  |  |  |
| Peripheral artery disease | 0.74 (0.384–1.328) | 0.338 | -0.958 |  |  |  |
| Diabetes mellitus |  |  |  |  |  |  |
| DM not on insulin vs. No DM | 0.854 (0.674–1.075) | 0.183 | -1.331 |  |  |  |
| DM on insulin vs. No DM | 0.479 (0.237–0.879) | **0.026** | -2.226 |  |  |  |
| COPD | 1.49 (0.985–2.217) | 0.053 | 1.934 |  |  |  |
| Dyslipidaemia | 0.681 (0.543–0.849) | **0.001** | -3.374 | 0.756 (0.594–0.956) | **0.021** | -2.309 |
| Chronic hepatic disease | 1.052 (0.675–1.598) | 0.817 | 0.232 |  |  |  |
| History of stroke | 1.32 (0.974–1.771) | 0.069 | 1.821 |  |  |  |
| History of ischaemic stroke | 1.445 (1.039–1.989) | **0.026** | 2.228 | 1.513 (1.003–2.274) | **0.047** | 1.987 |
| History of TIA | 1.261 (0.705–2.167) | 0.416 | 0.814 |  |  |  |
| History of ischaemic stroke or TIA | 1.369 (1.017–1.827) | **0.035** | 2.106 |  |  |  |
| Previous intracranial haemorrhage | 0.64 (0.147–1.968) | 0.484 | -0.7 |  |  |  |
| Previous major bleeding | 0.648 (0.217–1.58) | 0.381 | -0.876 |  |  |  |
| Previous major or CRNM bleeding | 0.836 (0.332–1.844) | 0.678 | -0.416 |  |  |  |
| VKA use | 0.784 (0.61–1.001) | 0.054 | -1.925 | 0.805 (0.616–1.045) | 0.108 | -1.608 |
| Other NOACs use | 0.898 (0.746–1.081) | 0.258 | -1.13 |  |  |  |
| Antiarrhythmics use | 0.989 (0.822–1.19) | 0.907 | -0.116 |  |  |  |
| Antiplatelet use | 1.668 (1.32–2.101) | **<0.001** | 4.319 | 1.628 (1.26–2.095) | **<0.001** | 3.759 |
| NASAIDs use | 1.054 (0.45–2.455) | 0.903 | 0.121 |  |  |  |

AF, Atrial fibrillation; BMI, Body mass index; Calc, calculated; CHF, Congestive heart failure; COPD, chronic obstructive pulmonary disease; CRNM, clinically relevant non–major; DM, Diabetes mellitus; ICH, intracranial haemorrhage; LVEF, left ventricular ejection fraction; NSAID, Non–steroidal anti–inflammatory drugs; OD, Odds Ratio; TIA, transient ischaemic attack; VKA, vitamin K antagonist; SD, standard deviation.

**Table S5** Factors associated edoxaban treatment not in line with 30 mg package insert recommendation in AF patients

|  | **Univariable** | | | **Multivariable** | | |
| --- | --- | --- | --- | --- | --- | --- |
|  | **OR (95% CI)** | **P value** | **Wald Z** | **OR (95% CI)** | **P value** | **Wald Z** |
| Male gender | 1.777 (1.405–2.247) | **<0.001** | 4.797 |  |  |  |
| Age |  |  |  |  |  |  |
| 65–74 vs. <65 years | 0.818 (0.587–1.148) | 0.239 | -1.178 |  |  |  |
| 75–84 vs. <65 years | 0.494 (0.351–0.699) | **<0.001** | -4.019 |  |  |  |
| ≥85 vs. <65 years | 0.422 (0.241–0.712) | **0.002** | -3.141 |  |  |  |
| Weight |  |  |  |  |  |  |
| <60 vs. 60–90 kg | 0.236 (0.185–0.301) | **<0.001** | -11.597 | 0.181 (0.137-0.238) | **<0.001** | -12.2 |
| ≥90 vs. 60–90 kg | 0.748 (0.107–3.503) | 0.73 | -0.345 | 1.085 (0.149-5.403) | 0.925 | 0.094 |
| BMI |  |  |  |  |  |  |
| Underweight vs. normal | 0.305 (0.143–0.576) | **0.001** | -3.37 |  |  |  |
| Overweight vs. normal | 1.446 (1.055–1.963) | **0.02** | 2.33 |  |  |  |
| Obesity vs. normal | 0.955 (0.218–2.989) | 0.944 | -0.071 |  |  |  |
| Economic region |  |  |  |  |  |  |
| Middle | 0.708 (0.545–0.914) | **0.009** | -2.626 | 0.653 (0.492–0.863) | **0.003** | -2.977 |
| Northeast | 0.842 (0.485–1.394) | 0.52 | -0.643 | 0.804 (0.442–1.406) | 0.459 | -0.74 |
| West | 0.667 (0.428–1.01) | 0.064 | -1.853 | 0.663 (0.412–1.038) | 0.08 | -1.748 |
| Smoking |  |  |  |  |  |  |
| Current vs. never | 1.943 (1.188–3.096) | **0.006** | 2.73 |  |  |  |
| Formerly vs. never | 1.485 (0.968–2.223) | 0.062 | 1.87 |  |  |  |
| Alcohol abuse | 2.233 (1.029–4.586) | **0.033** | 2.131 |  |  |  |
| Creatinine Clearance (Cockcroft-Gault) |  |  |  |  |  |  |
| [50,80) vs. ≥80 mL/min | 0.718 (0.47–1.113) | 0.13 | -1.513 |  |  |  |
| [30,50) vs. ≥80 mL/min | 0.56 (0.367–0.869) | **0.008** | -2.642 |  |  |  |
| <30 vs. ≥80 mL/min | 0.24 (0.111–0.482) | **<0.001** | -3.851 |  |  |  |
| Renal impairment |  |  |  |  |  |  |
| Stage G2 vs. Stage G1 | 0.804 (0.392–1.755) | 0.565 | -0.575 | 0.94 (0.429–2.19) | 0.881 | -0.15 |
| Stage G3 vs. Stage G1 | 0.627 (0.316–1.331) | 0.198 | -1.287 | 0.827 (0.381–1.916) | 0.643 | -0.464 |
| ≥Stage G4 vs. Stage G1 | 0.322 (0.142–0.756) | **0.007** | -2.681 | 0.388 (0.152–1.026) | 0.051 | -1.952 |
| CHA_2_DS_2_-VASc score |  |  |  |  |  |  |
| [2–3] vs. <2 | 0.729 (0.494–1.091) | 0.116 | -1.57 | 0.578 (0.364–0.925) | **0.021** | -2.309 |
| [4–5] vs. <2 | 0.515 (0.342–0.784) | **0.002** | -3.148 | 0.353 (0.208–0.602) | **<0.001** | -3.852 |
| ≥6 vs. <2 | 0.293 (0.14–0.571) | **0.001** | -3.458 | 0.157(0.064–0.366) | **<0.001** | -4.178 |
| HAS-BLED score |  |  |  |  |  |  |
| 2 vs. <2 | 0.597 (0.442–0.809) | **0.001** | -3.359 |  |  |  |
| ≥3 vs. <2 | 0.656 (0.440–0.971) | **0.036** | -2.091 |  |  |  |
| High risk of stroke or bleeding | 0.615 (0.483–0.781) | **<0.001** | -3.975 |  |  |  |
| High bleeding risk | 0.731 (0.577–0.923) | **0.009** | -2.619 | 0.559 (0.393–0.795) | **0.001** | -3.238 |
| Frailty | 0.684 (0.433–1.042) | 0.089 | -1.699 |  |  |  |
| Risk of fall | 0.516 (0.11–3.672) | 0.435 | -0.781 |  |  |  |
| LVEF <40 % | 0.523 (0.227–1.052) | 0.093 | -1.682 | 0.606 (0.251–1.299) | 0.227 | -1.208 |
| Hypertension | 1.404 (1.078–1.844) | **0.013** | 2.482 | 1.808 (1.296-2.544) | **0.001** | 3.448 |
| History of heart failure | 0.634 (0.439–0.896) | **0.012** | -2.504 |  |  |  |
| Coronary heart disease | 0.79 (0.623–0.999) | 0.05 | -1.957 |  |  |  |
| Valvular disease | 0.872 (0.529–1.377) | 0.573 | -0.564 |  |  |  |
| Peripheral artery disease | 0.392 (0.117–0.976) | 0.075 | -1.78 |  |  |  |
| Diabetes mellitus |  |  |  |  |  |  |
| DM not on insulin vs. No DM | 1.282 (0.949–1.717) | 0.099 | 1.647 |  |  |  |
| DM on insulin vs. No DM | 1.435 (0.764–2.55) | 0.237 | 1.182 |  |  |  |
| COPD | 1.329 (0.819–2.085) | 0.231 | 1.197 |  |  |  |
| Dyslipidaemia | 1.314 (1.006–1.707) | **0.042** | 2.031 |  |  |  |
| Chronic hepatic disease | 0.747 (0.379–1.353) | 0.364 | -0.908 |  |  |  |
| History of stroke | 0.823 (0.533–1.231) | 0.361 | -0.913 |  |  |  |
| History of ischaemic stroke | 0.917 (0.568–1.425) | 0.71 | -0.371 | 1.705 (0.976–2.905) | 0.054 | 1.925 |
| History of TIA | 1.667 (0.788–3.29) | 0.157 | 1.416 | 3.323 (1.437–7.344) | **0.004** | 2.909 |
| History of ischaemic stroke or TIA | 1.034 (0.679–1.533) | 0.87 | 0.164 |  |  |  |
| Previous ICH | 0.417 (0.023–2.189) | 0.405 | -0.832 |  |  |  |
| Previous major bleeding | 0.26 (0.014–1.279) | 0.192 | -1.306 |  |  |  |
| Previous major or CRNM bleeding | 0.218 (0.012–1.057) | 0.139 | -1.481 |  |  |  |
| VKA use | 0.887 (0.652–1.192) | 0.435 | -0.781 |  |  |  |
| Other NOACs use | 1.068 (0.846–1.348) | 0.58 | 0.553 |  |  |  |
| Antiarrhythmics use | 0.994 (0.787–1.255) | 0.962 | -0.047 |  |  |  |
| Antiplatelet use | 0.886 (0.627–1.23) | 0.482 | -0.703 |  |  |  |
| NASAIDs use | 1.657 (0.623–4.26) | 0.299 | 1.039 |  |  |  |
| P-gps use (Edoxaban Label listed) | 5.188 (1.686–16.185) | **0.004** | 2.89 |  |  |  |

AF, Atrial fibrillation; BMI, Body mass index; Calc, calculated; CHF, Congestive heart failure; COPD, chronic obstructive pulmonary disease; CRNM, clinically relevant non–major; DM, Diabetes mellitus; ICH, intracranial haemorrhage; LVEF, left ventricular ejection fraction; NSAID, Non–steroidal anti–inflammatory drugs; OD, Odds Ratio; P–gp, P–glycoprotein; TIA, transient ischaemic attack; VKA, vitamin K antagonist; SD, standard deviation.
